# HOTAIR requires epitranscriptomic modification to exert its pivotal epigenetic role in Epithelial to Mesenchymal Transition

**DOI:** 10.1038/s41419-025-08099-6

**Published:** 2025-10-24

**Authors:** Sabrina Garbo, Sara Minotti, Francesco Marocco, Luca Quattrocchi, Iris Di Silverio, Giulio Bontempi, Raffaele Strippoli, Marco Tripodi, Cecilia Battistelli

**Affiliations:** 1 Department of Molecular Medicine, Department of Excellence 2023-2027, Sapienza University of Rome, Rome, Italy; 2 Department of Epidemiology, Preclinical Research and Advanced Diagnostics, National Institute for Infectious Diseases L. Spallanzani, IRCCS, Rome, Italy

**Keywords:** Cell invasion, Long non-coding RNAs

## Abstract

While m6A epitranscriptomic modification has been shown to impact several mRNAs maturation, stability/degradation, nuclear/cytoplasm export and translation regulation, its impact on lncRNAs activity is yet largely uncharacterized. Here, we show that the silencing of the m6A writer METTL3 inhibits Epithelial to Mesenchymal Transition (EMT), morphological, migratory and invasive features of TGFβ-treated epithelial cells as well as of tumor cells. Building on previous evidence pinpointing the lncHOTAIR as a mandatory element for epithelial genes’ repression triggering EMT, here we uncover a dominant role of an epitranscriptomic modification on the epigenetic function of this lncRNA. Mechanistically, HOTAIR is m6A-modified on the interaction domains with both the master transcriptional factor of EMT SNAIL and the general chromatin modifier EZH2. This epitranscriptomic modification is necessary for the interaction between HOTAIR and SNAIL/EZH2 and in turn for HOTAIR-dependent epigenetic repression on SNAIL-targeted epithelial genes. Impairing m6A modification impedes the assembling of the tripartite SNAIL/HOTAIR/EZH2 complex and in turn blocks EMT accomplishment. Overall, we unveil that the epitranscriptomic modification m6A has a dominant role on the epigenetic function of a lncRNA.

## Introduction

The lncRNA HOTAIR (homeobox transcript antisense intergenic RNA) is a key regulator of chromatin dynamics and gene regulatory networks involved in a variety of pathophysiological processes (e.g., cell cycle regulation [1, 2], cellular metabolism [3–5], apoptosis [6], autophagy [7, 8], carcinogenesis [1], and epithelial to mesenchymal transition (EMT) [9, 10]). Regarding EMT, HOTAIR interacts with the sequence-specific transcription factor SNAIL and with the general chromatin modifier EZH2, catalytic subunit of Polycomb repressive complex 2 (PRC2), thus specifically conveying this ubiquitous chromatin modifier on SNAIL-target genes’ promoters and driving their transcriptional repression [11, 12] through an epigenetic reprogramming. These data disclosed HOTAIR as necessary for EMT as further demonstrated by a mutant form of HOTAIR representing the sole SNAIL-binding domain (HOTAIR-sbid) reported as dominant-negative in counteracting EMT and HCC (Hepatocellular Carcinoma) cells invasive and migratory abilities by interfering with the endogenous lncRNA [12].

Recently, it has been shown that HOTAIR is post-transcriptionally modified by the m6A writer METTL3 (Methyltransferase 3) and that site-specific epitranscriptomic modifications allow HOTAIR interaction with the YTHDC1 reader; notably, while YTHDC1 binding regulates HOTAIR stability, this interaction is not propaedeutic to its association with the chromatin causing gene repression and cell proliferation/invasion in breast cancer cells [13].

These data implement the growing research field of the epitranscriptomic modifications occurring on coding and non-coding RNAs, that influence biogenesis, stability, translation, interaction with proteins, compartmentalization and regulatory abilities on target genes [14, 15].

We here investigate on the impact of the m6A modification on HOTAIR interaction with DNA/RNA-binding proteins (i.e., SNAIL and EZH2) and, in turn, on HOTAIR-mediated epithelial gene repression in early stages of EMT.

Notably, two models of EMT cells (epithelial cells undergoing EMT in response to TGFβ treatment and mesenchymal tumor cells) silenced for METTL3 were found to recover an epithelial morphology, to decrease their migratory and invasive abilities, and to rescue the expression of epithelial markers despite high levels of SNAIL expression.

Mechanistically, this was found to depend on the m6A-dependent HOTAIR interaction with both SNAIL and EZH2, triggering H3K27me3 accumulation on specific SNAIL-target epithelial genes’ promoters and in turn their transcriptional silencing established through this epigenetic modification.

Moreover, the reported data indicate that m6A methylation profoundly influences HOTAIR structure, instrumental to its function.

## Materials and methods

### Cell culture conditions

Non-tumorigenic D3 murine hepatocytes [16] were grown on collagen I-coated dishes in RPMI-1640 supplemented with 10% fetal bovine serum (Gibco Life Technology, Monza, Italy), 50 ng/ml epidermal growth factor, 30 ng/ml insulin-like growth factor II (PeproTech Inc., Rocky Hill, NJ, USA), 10 μg/ml insulin (Roche, Mannheim, Germany) and antibiotics. Where reported, cells were treated with 2,5 ng/ml of TGFβ (PeproTech Inc., Rocky Hill, NJ, USA) for 72 h to induce EMT. SW480 colorectal cells were grown in DMEM supplemented with 10% fetal bovine serum (Gibco Life Technology, Monza, Italy) and antibiotics. All cell lines were tested for mycoplasma using the DAPI staining and the LookOut® Mycoplasma PCR Detection Kit (MP0035, Merck, Darmstadt, Germany). All cell lines were authenticated after thawing by morphology check, cell proliferation rate evaluation and species verification by PCR. Bacteria contamination was excluded.

### Lentiviral packaging, cell infection and transfection

Lentiviral particles were prepared as in [17]. Mouse Lentiviral vectors: SMARTvector Inducible Mouse Mettl3 mCMV-TurboGFP shRNA, SMIn NTC mCMV/GFP(Horizon, Dharmacon, Lafayette, Colorado, USA). Human Lentiviral Vectors as in [14].

The induction of shRNA was obtained by doxycycline treatment (2 μg/mL) for 48 h. For dose response experiments, cells were treated with different amounts of doxycycline (0,5 μg/mL, 1 μg/mL or 2 μg/mL).

For overexpression experiments, cells were transfected with FuGENE HD Transfection Reagent (E2311, Promega), according to the manufacturer’s protocol. Equal amounts of DNA (pTRACER or pTRACER-HOTAIR, pCMV6-METTL3 or pCMV6) were used. Cells were analyzed 48 h post-transfection.

### RNA extraction, reverse transcription, quantitative polymerase chain reaction

RNAs were extracted by ReliaPrep™ RNA Tissue Miniprep System (Promega, Madison, WI, USA) and reverse transcribed with iScriptTM c-DNA Synthesis Kit (Bio-Rad Laboratories Inc., Hercules, CA, USA). Quantitative polymerase chain reaction (RT-qPCR) analyses were performed according to MIQE guidelines [18]. cDNAs were amplified by qPCR reaction using GoTaq qPCR Master Mix (Promega, Madison, WI, USA). Relative amounts, obtained with 2(-ΔCt) method, were normalized with respect to the housekeeping genes (as indicated in figure legends).

For primer details see Table 1.

**Table 1:** List of primers used for meRIP, RIP and gene expression analysis.

Name	Sequence
mmu-METTL3 FW	GGACTCTGGGCACTTGGATT
mmu-METTL3 REV	GCACGGGACTATCACTACGG
mmu-GAPDH FW	CTGGTAAAGTGGATATTGTTGCCAT
mmu-GAPDH REV	TGGAATCATATTGGAACATGTAAACC
mmu-E-cadherin FW	CTACTGTTTCTACGGAGGAG
mmu-E-cadherin REV	CTCAAATCAAAGTCCTGGTC
mmu-HNF4a FW	TCTTCTTTGATCCAGATGCC
mmu-HNF4a REV	GGTCGTTGATGTAATCCTCC
mmu-SNAIL FW	CCACTGCAACCGTGCTTTT
mmu-SNAIL REV	CACATCCGAGTGGGTTTGG
mmu-HOTAIR FW	GCGCCAACGTAGACCAAAAG
mmu-HOTAIR REV	TACCGATGTTGGGGACCTCT
mmu-18S FW	ACGACCCATTCGAACGTCTG
mmu-18S REV	GCACGGCGACTACCATCG
hsa-E-cadherin FW	TACGCCTGGGACTCCACCTA
hsa-E-cadherin REV	CCAGAAACGGAGGCCTGAT
hsa-HNF4a FW	CATGGACATGGCCGACTACA
hsa-HNF4a REV	ATTGCCCATCGTCAACACCT
hsa-SNAIL FW	CACTATGCCGCGCTCTTTC
hsa-SNAIL REV	GCTGGAAGGTAAACTCTGGATTAGA
hsa-HOTAIR FW	CGGGACTTAGACCCTCAGGT
hsa-HOTAIR REV	GTTCCATTCCACTGCGAAGC
mmu-Sbid FW	GAAGACACGCACGGAGAAAG
mmu-Sbid REV	ACTGGGGTTTGTCTGGAGTT
mmu-Ezh2 bd FW	ACTTTGCTGCTGTGGAATGG
mmu-Ezh2 bd REV	TCTCTCCTTTTCTGCCTCTGG
mmu-HPRT1 FW	AGGACCTCTCGAAGTGTTGG
mmu-HPRT1 REV	TTGCAGATTCAACTTGCGCT
hsa-Sbid FW	GTCCTAGCTCGCCACATGAA
hsa-Sbid REV	TAAGAAGAGCAAGGAAGCCCC
hsa-Ezh2 bd FW	CATTCTGCCCTGATTTCCGG
hsa-Ezh2 bd REV	GGGGTGTTGGTCTGTGGAA
hsa-HPRT1 FW	ATCGCCAGTAAAATTATCAATG
hsa-HPRT1 REV	ACAAAACAGATAAAATTCTTAG
mmu-L34 FW	GGAGCCCCATCCAGACTC
mmu- L34 REV	CGCTGGATATGGCTTTCCTA
hsa-L32 FW	GGAGCGACTGCTACGGAAG
hsa-L32 REV	GATACTGTCCAAAAGGCTGGAA

### Protein extraction and western blot analysis

Cells were lysed in Laemmli buffer, subsequently the proteins were resolved on sodium dodecyl sulfate-polyacrylamide gel electrophoresis and transferred to nitrocellulose membrane 0.45 µm (162-0115; Bio-Rad Laboratories, Hercules, CA). The following primary antibodies were used for immunoblotting: α-METTL3 (195352 Abcam, Cambridge, UK), α-HNF4α (MA1-199 Thermo Fisher Scientific, Waltham, MA, USA), α-E-CADHERIN (610182 BD transduction laboratories, Franklin Lakes, New Jersey), α-Snail (L70G2, Cell Signaling Technology, Danvers, MA, USA) and α-GAPDH (MAB-374 Merck, Darmstadt, Germany), used as a loading control. The immune complexes were detected with horseradish peroxidase-conjugated species-specific secondary antiserum: (α-Rabbit 172-1019 and α-Mouse 170-6516 Bio-Rad Laboratories, Hercules, CA), followed by chemiluminescence reaction (Bio-Rad Laboratories, Hercules, CA). Densitometric analysis of protein expression was performed by using the Fiji-Image J image processing package.

### RNA dot blot

RNA samples were incubated with RNA buffer (formamide, MOPS, and 37% formaldehyde) at 65 °C for 5 min and then 20X Saline Sodium Citrate Solution was added. 100 ng of RNAs were spotted onto a nylon membrane (Hybond-N, RPN.203 N., Amersham, Freiburg, Germany) by Bio-dot apparatus (Bio-Rad Laboratories, Hercules, CA) and RNA was UV-crosslinked (2 pulses, 30 s, 1200 μjoules in a UV Stratalinker 1800, Stratagene, La Jolla, CA, USA) to the membrane. The membrane was incubated with 0.02% Methylene blue (to label total RNA) and with anti-m6A antibody (202 003, Synaptic System, Goettingen, Germany) (to label m6A-modified RNA) at 4 °C, over-night. The immune complexes were detected as in Western-blot analysis.

### Immunofluorescence

Cells were methanol-fixed, incubated with 3% BSA in PBS (for 1 hour), and then with anti-E-cadherin (610182 BD transduction laboratories, Franklin Lakes, New Jersey), or anti-KI67 (SAB4501880, Merck, Darmstadt, Germany). Goat anti-Mouse FITC-conjugated secondary antibody (31992, Thermo Fisher Scientific, Waltham, MA, USA), Donkey anti-Rabbit 594-conjugated Secondary Antibody (A21207, Thermo Fisher Scientific, Waltham, MA, USA) were used. Nuclei were stained with DAPI (4′,6-diamidino-2-phenylindole; 268298; Calbiochem Merck, Germany). Images were examined with Nikon Microphot-FXA microscope (Nikon Corporation, Japan) equipped with a CCD camera. Digital images were acquired with a 20X objective by Nikon NIS-elements software (Nikon Corporation, Japan) and scale bars were added as indicated in each figure.

### Scratch assay

Cell lines were maintained in culture medium (as above) until reaching 100% confluence, then shifted to serum-depleted culture medium to inhibit cell proliferation; a scratch wound was created on the cell layer using a micropipette tip. Micrographs were taken at time 0 and 48 h after the scratch, with a 4X objective. Micrographs were captured with Kamera ToupCam (E3ISPM 6300B, color, CMOS, 1/1.8”, 2,4 µm, 59 fps, 6.3 MP, USB 3.0, Torino, Italy) and scale bar were added by the camera software Image View. Cell-devoid areas at time 0 and 48 h after the scratch were quantified through Fiji-Image J image processing package.

### Invasion assay

For transwell invasion assays, 8 μm pore 24-well cell culture plates (Corning Inc, NY, USA), coated with type I collagen (0.1 mg/ml; Upstate Biotechnology, CA, USA) were used. Equal numbers of cells were plated in the upper chamber in serum-free medium, while the lower chamber medium was supplemented with 20% FBS as a chemoattractant. Cells were fixed with 100% MetOH, stained with Giemsa’s solution, and manually counted in random microscopic fields. Micrographs were captured with a 4X objective using Kamera ToupCam (E3ISPM 6300B, color, CMOS, 1/1.8”, 2,4 µm, 59 fps, 6.3 MP, USB 3.0, Torino, Italy) and scale bar were added by the camera software Image View.

### Methylated RNA immunoprecipitation (MeRIP)

meRIP was performed in accordance to [19]. Antibody against m6A (202 003; Synaptic System, Goettingen, Germany) or Normal Rabbit IgG (12–370; Merck, Darmstadt, Germany) was added to the RNA diluted in IP buffer (10 mM Tris [pH 7.5], 150 mM NaCl, 0.5% Nonidet P-40) and then incubated with protein A magnetic beads. RNA samples were eluted with elution buffer (5 mM Tris-HCl pH 7.5, 1 mM EDTA, 0.05% SDS), extracted using the phenol-chloroform method, purified, and then analyzed by qRT-PCR. Relative amounts, obtained with 2(-ΔCt) method, were normalized with respect to the Input sample and then represented as IP/IgG.

For primer sequences see Table 1. Efficiency of 91% for mmu-Sbid primers, 104,1% for mmu-Ezh2 bd primers, 108,8% for mmu-HPRT1 primers, 95,6% for hsa-Sbid primers, 92,6% for hsa-Ezh2 bd primers and 94,6% for hsa-HPRT1 primers.

### Bioinformatic structural predictions

Secondary structure predictions of RNA structure and secondary structure propensity was performed by CROSS (http://service.tartaglialab.com/update_submission/953530/6fdef887b8) [20]. RNA Secondary Structures (RSS) score was reported on the y-axis of the graphs in dependence on the nucleotide sequence reported on the x-axis.

### Cross-linked RNA immunoprecipitation (CLIP)

CLIP was performed as reported in [21] starting from 1 mg of cleared lysate previously cross-linked (1 pulse, 30 s, 8000 μjoules in a UV Stratalinker 1800, Stratagene, La Jolla, CA, USA). Immunoprecipitated RNA was reverse transcribed and used in real-time polymerase chain reaction (RT-qPCR) amplifications. List of primers is reported in Table 1. Primary antibodies for IP were: anti-EZH2 39901 (Active motif, Waterloo, Belgium), anti-Snail AF3639 (R&D systems, Minneapolis, MN, USA) and as negative control Normal Rabbit IgG 12370 (Merck, Darmstadt, Germany) or Normal Goat IgG AB-108-C (R&D systems, Minneapolis, MN, USA).

### Co-immunoprecipitation

Cells were lysed in 50 mM Tris-HCl (pH 8.0), 150 mM NaCl, 5 mM EGTA (pH 8.0), 50 mM NaF (pH 8.0), 10% glycerol, 1.5 mM MgCl2, 1% Triton X-100 containing protease and phosphatase inhibitors (complete EDTA-free; Roche Applied Science, Mannheim Germany) and protein concentrations determined by Bradford method. One milligram of cell lysates, after preclearing with protein G-sepharose (GE Healthcare, Little Chalfont, Buckinghamshire, UK), was incubated with 5 μg of anti-Snail AF3639 (R&D systems, Minneapolis, MN, USA) and as negative control Normal Goat IgG AB-108-C (R&D systems, Minneapolis, MN, USA). The complexes were incubated for 3 h with protein G-sepharose. Immune complexes were washed (as in [12]), eluted and denatured in Laemmli buffer. Proteins from either cell lysates or immunoprecipitation were resolved on sodium dodecyl sulfate-polyacrylamide gel electrophoresis and transferred to nitrocellulose membrane (162-0115; Bio-Rad Laboratories, Hercules, CA, USA). Blots were probed with primary anti-EZH2 05-1319 (Merck, Darmstadt, Germany) or anti-Snail L70G2 (Cell Signaling Technology Inc., Danvers, MA, USA) and immune complexes were detected with horseradish peroxidase-conjugated species-specific secondary antiserum (Bio-Rad Laboratories, Hercules, CA, USA), followed by enhanced chemiluminescence reaction (Bio-Rad Laboratories, Hercules, CA, USA).

### Chromatin immunoprecipitation (ChIP) analysis

ChIP analysis was performed by using 5 μg of the specific antibody (H3K27me3; 07-449; Merck, Darmstadt, Germany) or the negative control normal rabbit IgG (Merck, Darmstadt, Germany), of the anti-Snail AF3639 (R&D systems, Minneapolis, MN, USA) or the negative control Normal Goat IgG AB-108-C (R&D systems, Minneapolis, MN, USA), as previously reported [11]. The DNA was extracted with phenol-chloroform solution, precipitated with NaCl and isopropanol and resuspended in 50 μl of water, then used in the downstream qPCR analyses (primer pairs details are listed in Table 2).

**Table 2:** List of primers used for ChIP analysis.

Name	Sequence
mmu-HNF4 ebox 1 FW	GGAGATGGAAACTGAGGCTTG
mmu-HNF4 ebox 1 REV	CAGCGGTTGGATATCTCTTG
mmu-HNF4 ebox 2 FW	CGGTTCCCAAAGCATGTGAC
mmu-HNF4 ebox 2 REV	ATAAAGCTGTCCTGGGTCGC
mmu-e-cad ebox FW	GAACGACCGTGGAATAGGAA
mmu-e-cad ebox REV	CTCCCACACCAGTGAGCAG
mmu-rpl30 FW	TAAGGCAGGAAGATGGTGG
mmu-rpl30 REV	CAGTGTGCTCAAATCTATCC
hsa-HNF4 ebox FW	CAAGCAGGTGGTGAGATCC
hsa-HNF4 ebox REV	CGTCTCCTCTGGTCTCCTTC
hsa-e-cad ebox FW	GGCAAGACAGAGCGAGAC
hsa-e-cad ebox REV	TCGAACTCCTGGGCTGAA
hsa-rpl30 FW	GCAGGAAGATGGTGGCCGCAA
hsa-rpl30 REV	AGTCTGCTTGTACCCCAGGACGT

### Statistical analysis

Paired t-test was applied by using GraphPad Prism version 8.00 (GraphPad software, San Diego, CA, USA; http://www.graphpad.com). A *P*-value (*P*) < 0.05 was considered statistically significant (*P < 0.05; **P < 0.01 and ***P < 0.001). Data were obtained from independent experiments and expressed as means ± SD. Number of experimental replicates, statistical methods/tests and the definition of error bars are reported in each Figure legend.

## Results

### m6A modification is required in EMT induction

**Fig. 1:**
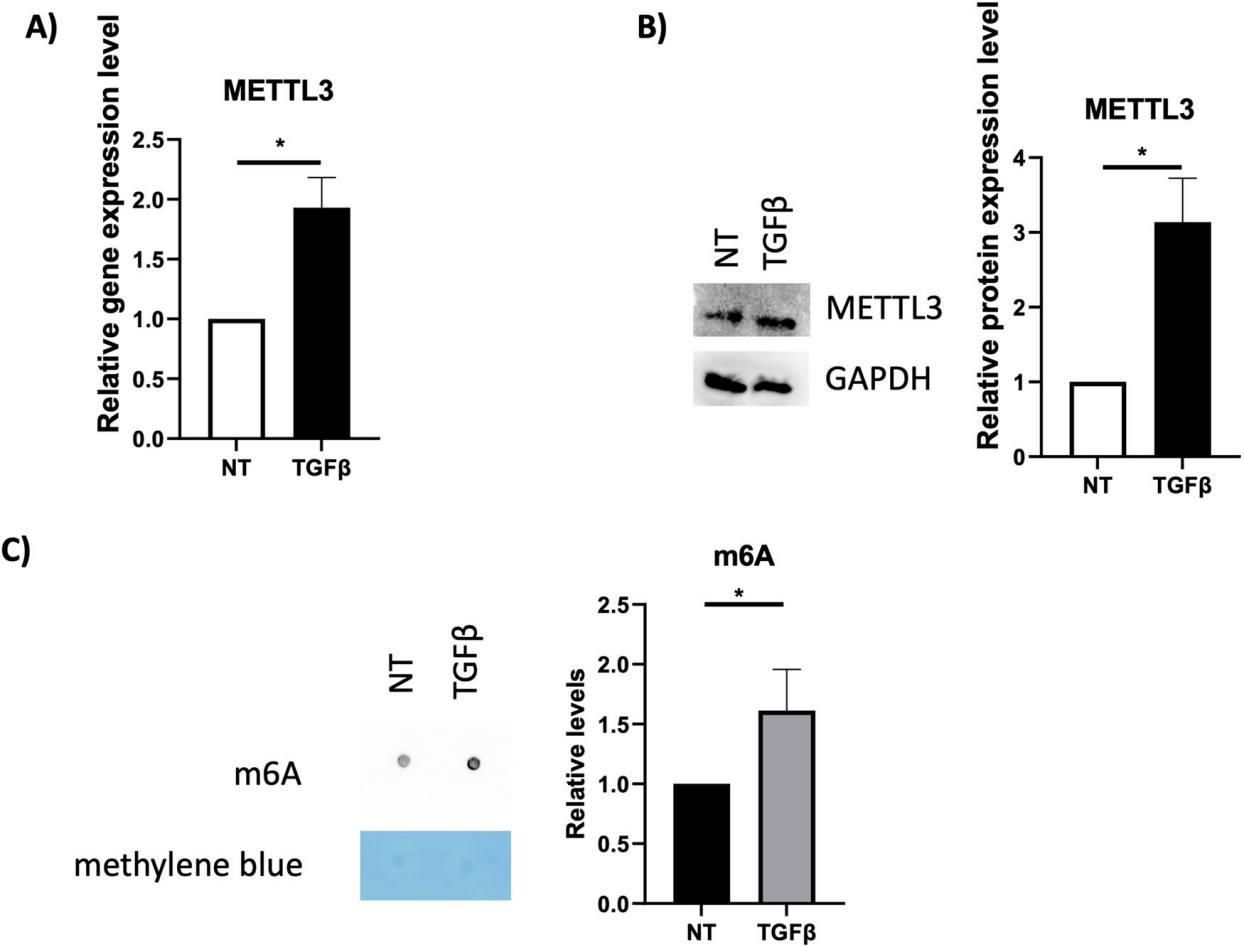
TGFβ-mediated m6A upregulation. **A** Relative expression level of METTL3 mRNA in murine non-tumorigenic hepatocytes untreated or treated with TGFβ (NT or TGFβ), analyzed by RT-qPCR. Data have been normalized with respect to GAPDH expression and are shown as the mean ± SD of three independent experiments. NT arbitrary value 1. **B** (Left) Western-blot analysis for METTL3 on protein extracts from hepatocytes treated with TGFβ (TGFβ) and relative untreated control (NT). GAPDH has been used as loading control. The figure is representative of three independent experiments. (Right) Densitometric analysis of Western-blot signals. Data are shown as the mean ± SD of three independent experiments. **C** (Left) RNA dot blot on total RNA from hepatocytes treated with TGFβ (TGFβ) or left untreated (NT) as control. Methylene blue has been used as loading control. The figure is representative of three independent experiments. (Right) Densitometric analysis of Dot-blot signals. Data are shown as the mean ± SD of three independent experiments. **A**–**C** Data are considered statistically significant with *p* < 0.05 (two-tailed paired *t*-test). *p < 0.05.

To address the involvement of m6A modification in TGFβ-induced EMT, a non-tumorigenic hepatic cell line has been silenced for METTL3 through an shRNA-based approach. As shown in Fig. 1A–C, TGFβ treatment inducing EMT increases both METTL3 and m6A abundance as measured by qPCR, Western Blot analysis and RNA dot blot, respectively.

Notably, the genetic silencing of METTL3 highlights its requirement for the induction of EMT.

**Fig. 2:**
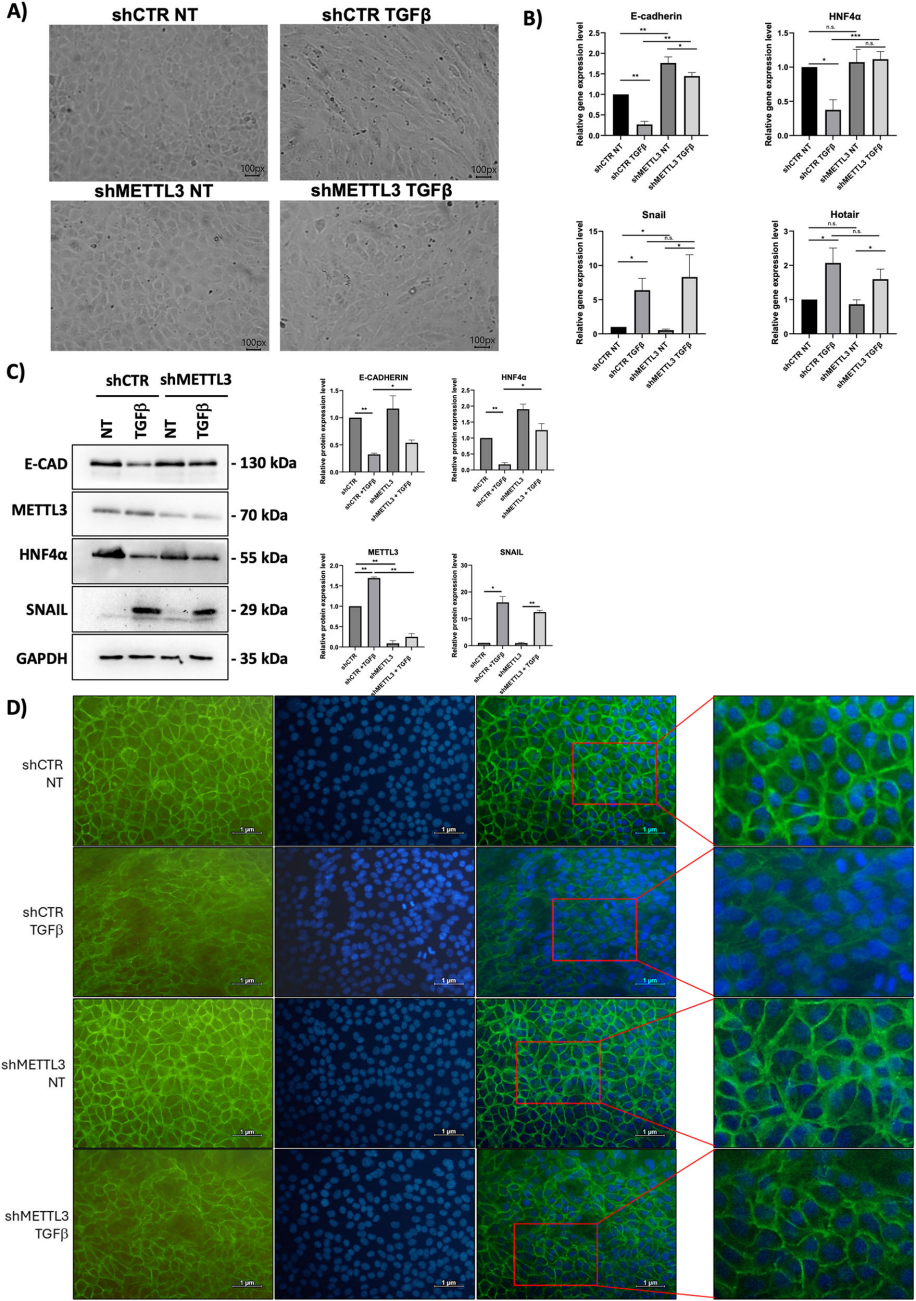
m6A modification is required in EMT induction. **A** Phase contrast micrographs for the analysis of murine non-tumorigenic hepatocytes expressing shCTR or shMETTL3 (shRNAs) and treated with TGFβ or left untreated (NT) as indicated. Scale bars are reported. **B** Relative expression level of the indicated mRNAs in hepatocytes as in (**A**), analyzed by RT-qPCR. Data have been normalized with respect to 18S expression and are shown as the mean ± S.E.M. of four independent experiments. NT arbitrary value 1. **C** (Left) Western-blot analysis for the indicated proteins on extracts from hepatocytes as in (**A**). GAPDH has been used as loading control. The figure is representative of three independent experiments. (Right) Densitometric analysis of Western-blot signals. Data are shown as the mean ± S.E.M. of three independent experiments. **D** Immunofluorescence detection of E-cadherin in hepatocytes as in (**A**). DNA-DAPI (blue), E-cadherin (green). (Right) Magnification of merged figures is reported. Scale bars are reported. **B**, **C** Data are considered statistically significant with *p* < 0.05 (paired *t*-test). *p* value: * < 0.05; ** < 0.01; *** < 0.001.

In fact, morphological analysis of hepatocytes demonstrates that while METTL3-expressing cells acquire a spindle-like morphology in response to TGFβ treatment, METTL3-silenced hepatocytes maintain a more epithelial-like cobblestone shape, representative of epithelial cells (Fig. 2A). This is in line with a molecular profile where the reduction of the expression of the epithelial/hepatic markers E-cadherin and HNF4α occurring in response to TGFβ treatment in hepatocytes is not observed upon METTL3 depletion (Fig. 2B, top).

Moreover, to address a quantitative connection between METTL3 levels and HOTAIR functional impairment, a METTL3 silencing with different concentrations of doxycycline has been performed. By means of this protocol the induction of shRNA expression and METTL3 silencing follow the same trend of doxycycline dose and correlates with the EMT functional impairment as assessed by E-cadherin and HNF4α expression rescue (Supplementary Figure 1).

Mechanistically, the EMT impairment appears due to HOTAIR methylation directly as demonstrated by rescue experiments in hepatocytes undergoing EMT and silenced for METTL3; residual METTL3 indeed is sufficient to methylate the overexpressed HOTAIR (Supplementary Fig. 2A). In other words, although the transfected HOTAIR prevents methylation as the endogenous transcript, a significant level of HOTAIR methylation is detectable by meRIP assay (Supplementary Fig. 2B) and the impact on the EMT markers expression levels results significant (Supplementary Fig. 2C).

Conversely, with respect to the mesenchymal markers Snail and HOTAIR, their expression in response to TGFβ is not significantly modulated by METTL3 depletion (Fig. 2B, bottom). The gene expression profile matches with the relative protein levels and with E-cadherin localization at the cell membrane (Fig. 2C and D respectively).

On the other hand, untreated non tumorigenic hepatocytes do not significantly modulate epithelial genes expression (Fig. 2B–D) thus suggesting the specific involvement of HOTAIR; indeed, previous data demonstrated that in the absence of HOTAIR, Snail loses its repressive activity failing to trigger EMT [11]. To further dissect the role of METTL3, its overexpression was carried out and appears insufficient to promote EMT (Supplementary Fig. 3); indeed, in this condition, the pivotal elements for EMT program SNAIL and HOTAIR are negligibly expressed. Overall, EMT induction requires not only SNAIL/HOTAIR but also METTL3.

Similar results have been obtained in SW480 colorectal cancer cells, EMT-like cells expressing high levels of HOTAIR and Snail.

**Fig. 3:**
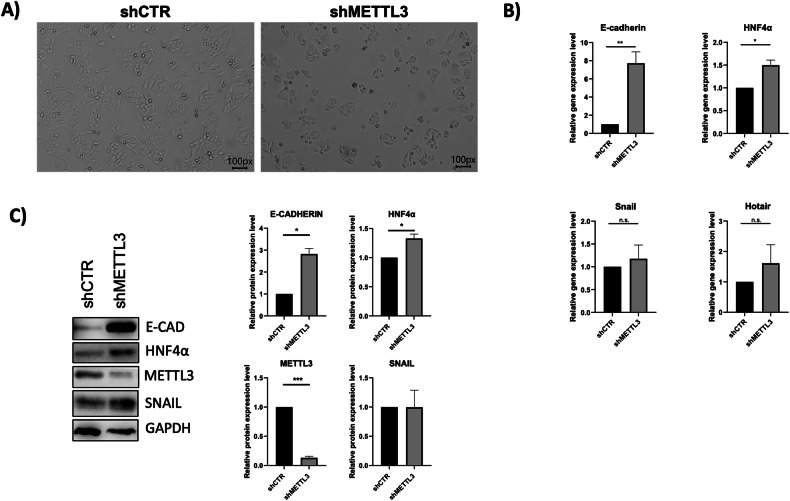
m6A modification is required in mesenchymal cancer cells. **A** Phase contrast micrographs for the analysis of SW480 cells expressing shCTR or shMETTL3 as indicated. Scale bars are reported. **B** Relative expression level of the indicated mRNAs in SW480 cells as in (**A**), analyzed by RT-qPCR. Data have been normalized with respect to L32 expression and are shown as the mean ± S.E.M. of four independent experiments. NT arbitrary value 1. **C** (Left) Western-blot analysis for the indicated proteins on extracts from SW480 cells as in (**A**). GAPDH has been used as loading control. The figure is representative of three independent experiments. (Right) Densitometric analysis of Western-blot signals. Data are shown as the mean ± S.E.M. of three independent experiments. **B**, **C** Data are considered statistically significant with *p* < 0.05 (paired *t*-test). *p* value: * < 0.05; ** < 0.01; *** < 0.001.

As shown in Fig. 3A, cells undergo a morphologic change, and the levels of epithelial markers increase in METTL3-silenced cells while not significant modulations are observed for Snail and HOTAIR at transcriptional levels (Fig. 3B). Again, gene expression profile matches with the relative protein levels showing a rescue of E-cadherin and HNF4α expression in silenced cells (Fig. 3C).

**Fig. 4:**
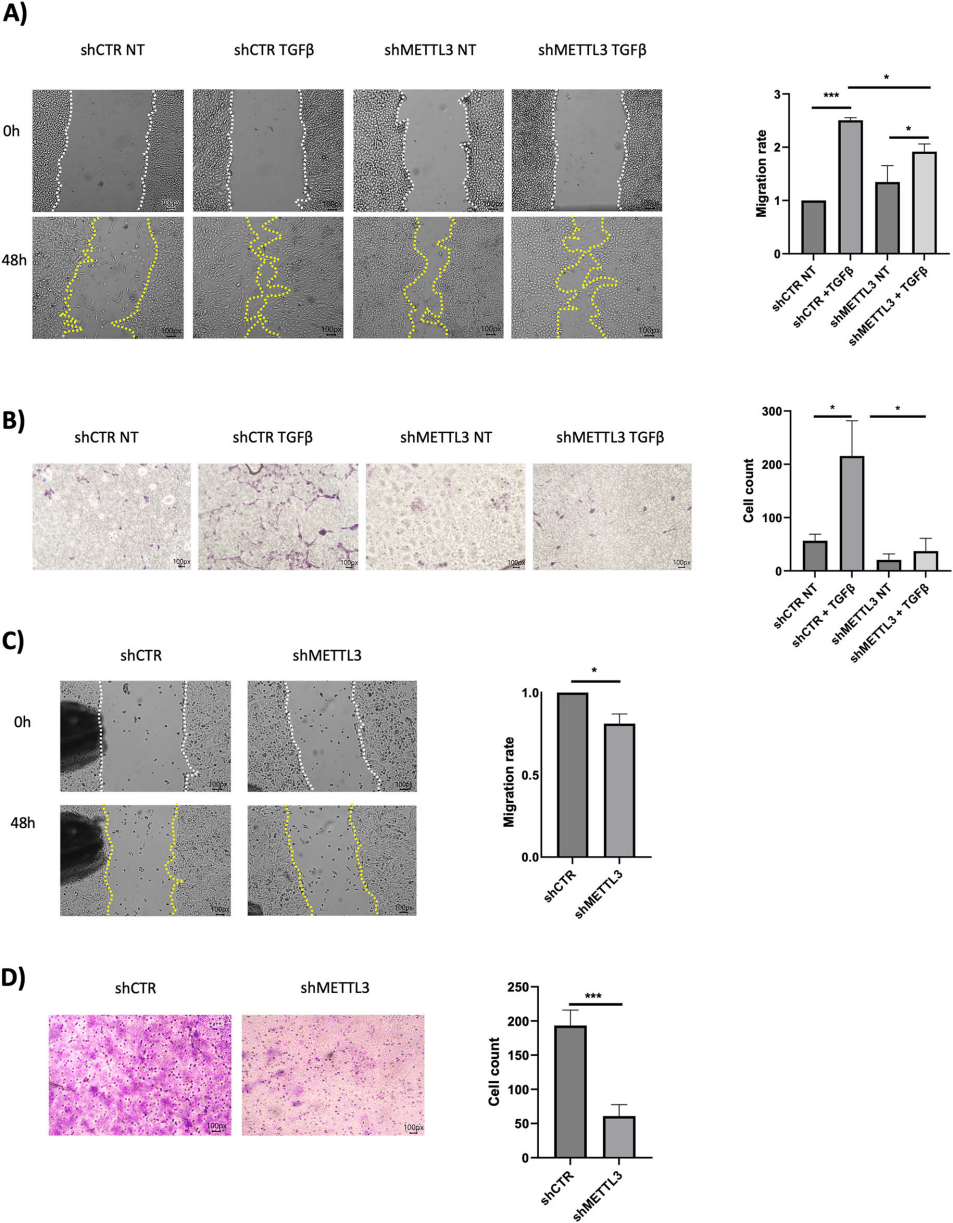
m6A modification is required for cell migratory and invasive abilities during EMT. **A** (Left) Scratch assay at the indicated time in murine non-tumorigenic hepatocytes expressing shCTR or shMETTL3, treated with TGFβ or left untreated (NT). The figure is representative of three independent experiments. For each experimental replicate, the same randomly chosen field has been monitored over time. (Right) Quantification of migration, data are shown as the mean ± SD of three independent experiments. **B** (Left) Invasion assay in murine non-tumorigenic hepatocytes as in (**A**). The figure is representative of three independent experiments. (Right) Quantification of invasion, data are shown as the mean ± SD of three independent experiments. **C** (Left) Scratch assay at the indicated time in SW480 cells expressing shCTR or shMETTL3. The figure is representative of three independent experiments. For each experimental replicate, the same randomly chosen field has been monitored over time. (Right) Quantification of migration, data are shown as the mean ± SD of three independent experiments. **D** (Left) Invasion assay in SW480 cells as in (**C**). The figure is representative of six independent experiments. (Right) Quantification of invasion, data are shown as the mean ± SD of six independent experiments. **A**–**D** Data are considered statistically significant with *p* < 0.05 (two-tailed paired *t*-test). *p* value: * < 0.05; *** < 0.001.

Functional assays extend the conclusion of a METTL3-dependent EMT induction in both cell models. Indeed, as shown in Fig. 4, migratory (assessed by scratch test, in the absence of proliferation as in Supplementary Fig. 4) (Fig. 4A, C) and invasive (assessed by transwell-invasion assay) (Fig. 4B, D) abilities are significantly impaired upon METTL3 silencing.

Overall, these data highlight a role for epitranscriptomics in the EMT dynamic process.

### HOTAIR methylation conditions its secondary structure

In order to provide a molecular mechanism of the described readout and in the light of the widespread distribution of m6A modification, the exploration of epitranscriptomic modifications was focused on transcripts previously described as molecular players, required in EMT.

HOTAIR has been reported as essential for the EMT induction and has been described as m6A modified in different cancer cell lines (HeLa, MDA-MB-231, MCF7) [13]; here this observation has been extended to the previously described experimental systems (i.e hepatocytes undergoing TGFβ-induced EMT and EMT-like colorectal cancer cells), both expressing detectable levels of HOTAIR.

Specifically, this modification has been explored on the previously defined SNAIL and EZH2-interacting sites at the 5’ of murine and human HOTAIR [11, 12, 22–24].

**Fig. 5:**
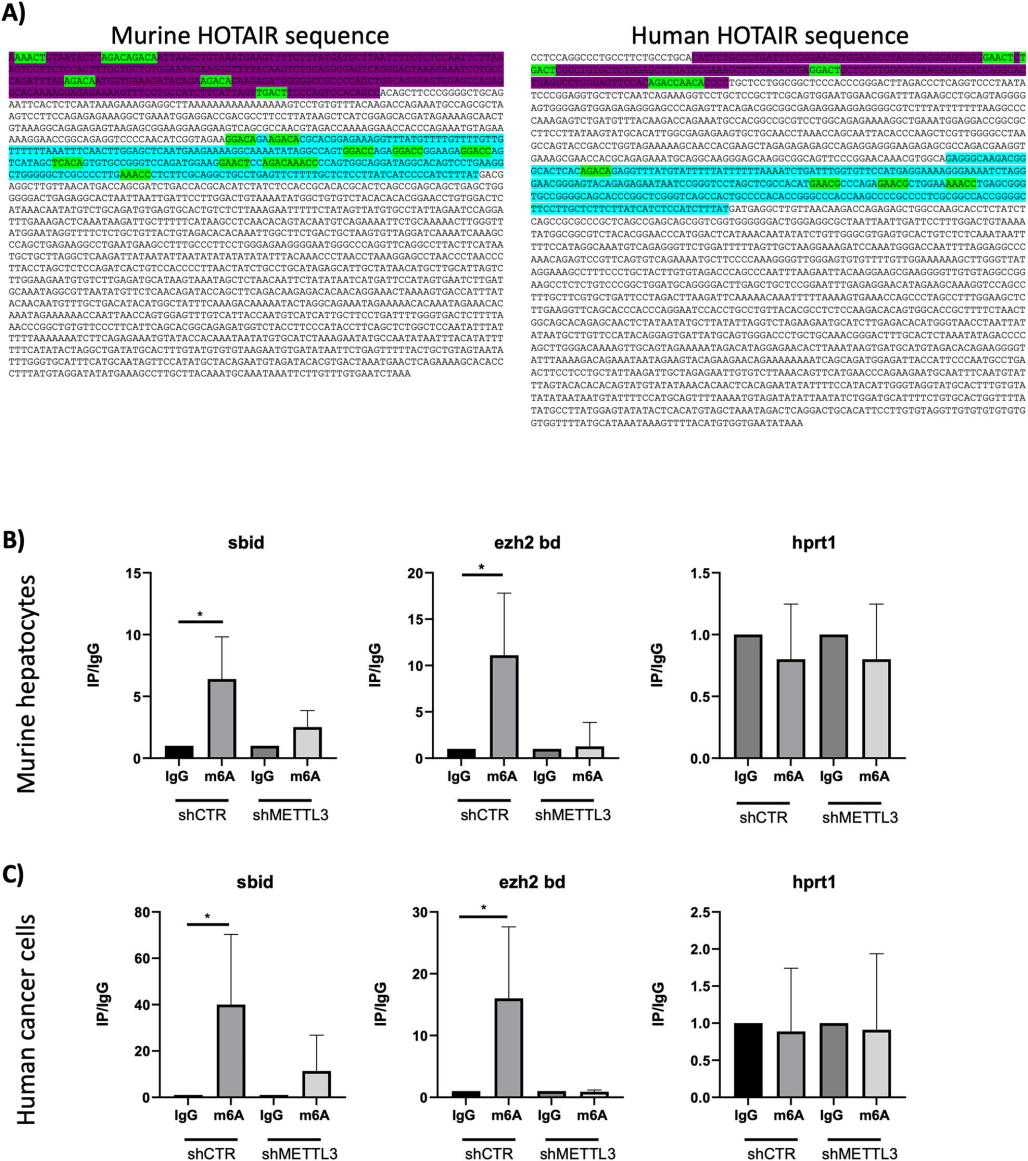
HOTAIR is m6A-modified on SNAIL and EZH2 binding domains. **A** Analysis of the presence of putative m6A sites on SNAIL and EZH2 binding domains along the murine and human HOTAIR sequence. EZH2 binding sites are highlighted in purple, Hotair SBID is highlighted in cyan and methylation sites are highlighted in green. **B** meRIP-qPCR analysis on HOTAIR SNAIL binding domain (sbid) and EZH2 binding domain (ezh2 bd) in murine non-tumorigenic hepatocytes expressing shCTR or shMETTL3 during TGFβ-induced EMT. Data are shown as the mean ± SD of three independent experiments and are represented as IP/IgG. HPRT1 was used as negative control. **C** meRIP-qPCR analysis on HOTAIR SNAIL binding domain (sbid) and EZH2 binding domain (ezh2 bd) in SW480 cells expressing shCTR or shMETTL3. Data are shown as the mean ± SD of three independent experiments and are represented as IP/IgG. HPRT1 was used as negative control. **B**–**C** IgG arbitrary value = 1. Data are considered statistically significant with *p* < 0.05 (two-tailed paired *t*-test). *p* value: * < 0.05.

The starting analysis of the sequence spanning these two domains highlighted the presence of several putative methylation sites [19] in both murine and human HOTAIR (Fig. 5A).

Moreover, m6A immunoprecipitation (meRIP) followed by qPCR amplification with specific primer sets has been carried out; notably, both regions result significantly methylated in both TGFβ-treated hepatocytes (Fig. 5B) and in SW480 cancer cells (Fig. 5C). As further control of meRIP specificity, the same analysis has been performed on both cell lines silenced for METTL3 (shMETTL3).

**Fig. 6:**
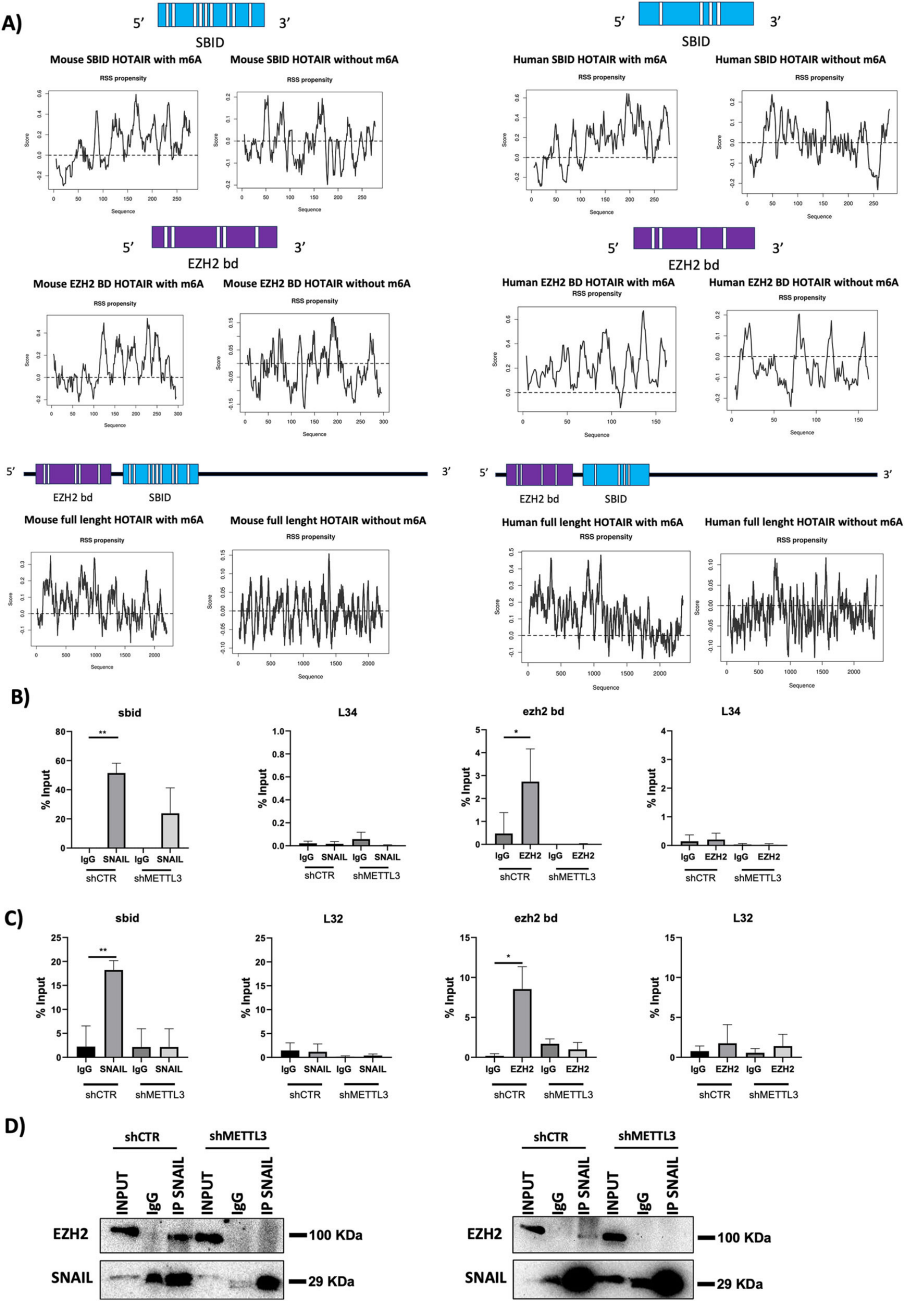
m6A modification is propaedeutic for HOTAIR-SNAIL and HOTAIR-EZH2 interaction. **A** Secondary structures propensity predicted by CROSS algorithm on the methylated or not methylated HOTAIR. (Top) HOTAIR-sbid. (Middle) HOTAIR-EZH2 bd. (Bottom) Full length HOTAIR. (Left) Murine HOTAIR secondary structures propensity. (Right) Human HOTAIR secondary structures propensity. On the y-axis the score for the prediction of secondary structures is represented along the RNA sequence reported on the x-axis. The SNAIL binding domain is labeled in cyan, the EZH2-binding domain is labeled in purple. White bars indicate the putative m6A sites. **B** (Left) RIP assays with anti-SNAIL or preimmune IgG on murine non-tumorigenic hepatocytes expressing shCTR or shMETTL3 during TGFβ-induced EMT. Data are reported as means ± SD of three independent experiments. (Right) RIP assays with anti-EZH2 or preimmune IgG on hepatocytes as in (Left). Data are reported as mean ± SD of four independent experiments. RNA levels in immunoprecipitates (IP) and IgG were determined by qRT-PCR. HOTAIR-*sbid*, HOTAIR-ezh2 bd and, as negative control, ribosomal L34 RNA, were reported as % Input. **C** (Left) RIP assays with anti-SNAIL or preimmune IgG on SW480 cells expressing shCTR or shMETTL3. Data are reported as mean ± SD of four independent experiments. (Right) RIP assays with anti-EZH2 or preimmune IgG on SW480 cells as in (Left). Data are reported as mean ± SD of three independent experiments. RNA levels in immunoprecipitates (IP) and IgG were determined by qRT-PCR. HOTAIR-*sbid*, HOTAIR-ezh2 bd and, as negative control, ribosomal L32 RNA, were reported as % Input. **D** Co-immunoprecipitation of EZH2 and SNAIL in murine hepatocytes (Left panels) and human colorectal cancer cells (Right panels). Western blot data are representative of three independent experiments. **B**–**C** Data are considered statistically significant with *p* < 0.05 (two-tailed paired *t*-test). *p* value: * < 0.05, ** < 0.01.

Furthermore, the possible impact of HOTAIR modification on its secondary structure has been explored by applying the CROSS algorithm [20]. As reported in Fig. 6A, the methylation of HOTAIR is predicted to influence the secondary structure of the murine and human lncRNA; it is appreciable a decrease of the RNA secondary structure (RSS) score (indicating the propensity of the RNA to acquire secondary structures) in the absence of m6A with respect to m6A presence.

Overall, these data disclose that HOTAIR is methylated in two different EMT models and specifically on two relevant domains of interaction with DNA/RNA-binding proteins.

### m6A modification defines HOTAIR function in EMT

Furthermore, the functional impact of the predicted m6A-dependent HOTAIR structure has been tested. Thus, cross-linked RNA Immunoprecipitation (CLIP) assay has been performed to evaluate the impact of m6A on HOTAIR interaction with SNAIL and EZH2 in TGFβ-treated hepatocytes (when both SNAIL and HOTAIR expression is induced) and in SW480 cancer cells (showing both SNAIL and HOTAIR constitutive expression). Interestingly, as shown in Fig. 6B, C, these interactions are abolished in response to METTL3 depletion (shMETTL3).

Moreover, co-immunoprecipitation experiments show that METTL3 depletion impairs the HOTAIR-mediated physical interaction between SNAIL and EZH2 (Fig. 6D).

**Fig. 7:**
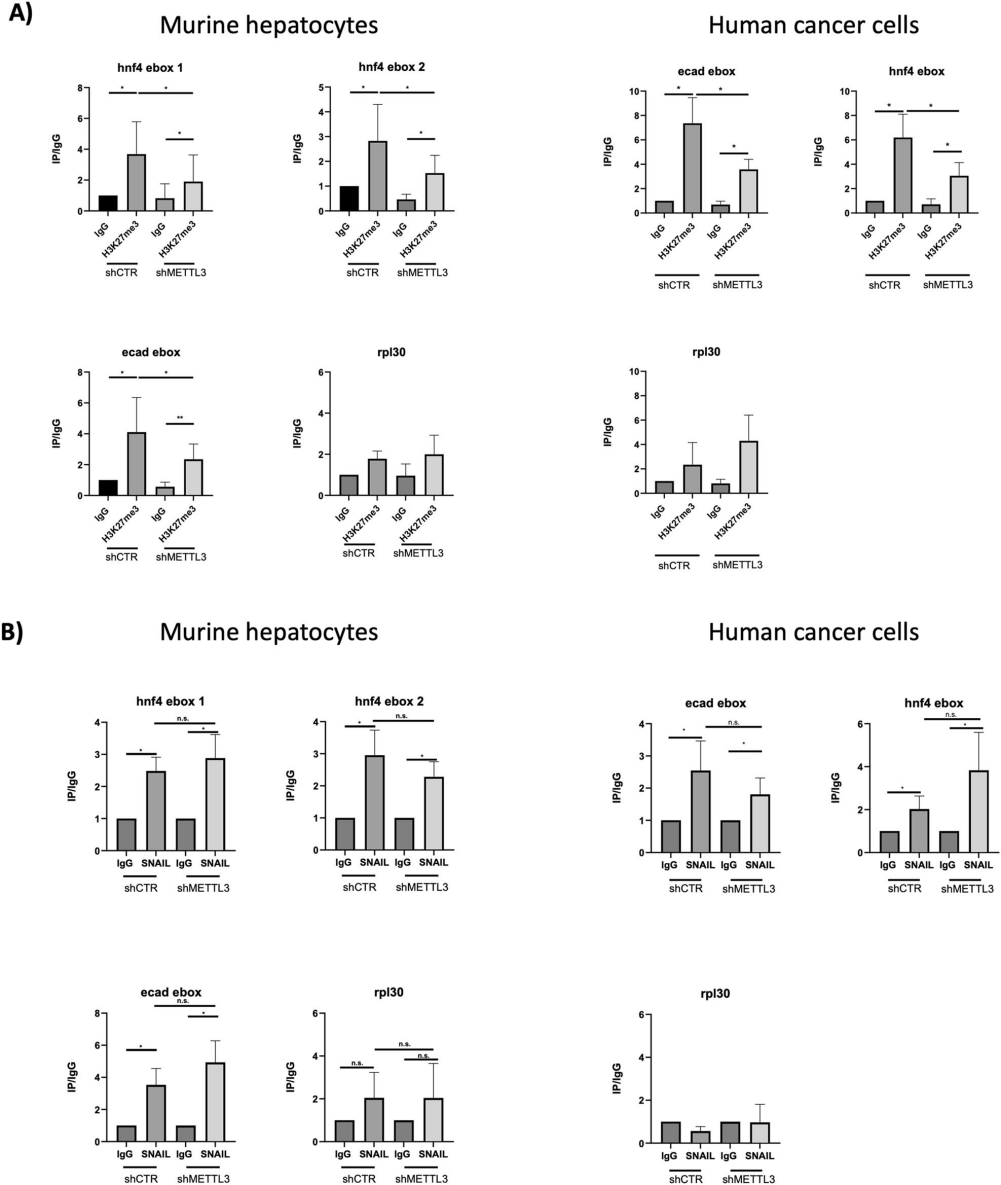
m6A is required for HOTAIR-dependent epigenetic modifications. **A** ChIP analysis for H3K27me3 on the indicated promoters in murine non-tumorigenic hepatocytes (Left panels) and in human colorectal cancer cells (Right panels). **B** ChIP analysis for SNAIL binding to the indicated promoters in murine hepatocytes (Left panels) and in human cancer cells (Right panels). Data are shown as IP/IgG and are reported as mean ± SD of independent experiments. IgG arbitrary value 1. Data are considered statistically significant with *p* < 0.05 (two-tailed paired *t*-test). *p* value: * < 0.05, ** < 0.01.

Finally, to gather insight into the molecular mechanism linking RNA methylation to epithelial gene repression in EMT, a Chromatin ImmunoPrecipitation (ChIP) approach for the repressive histone marker H3K27me3 (mediated by EZH2) has been performed on hepatocytes undergoing EMT as well as in SW480 cancer cells, both silenced for METTL3 and control. Specifically, the epigenetic modification was explored on the SNAIL-binding sites on the promoter regions of two main epithelial/hepatic SNAIL-target genes, E-cadherin and HNF4α. An enrichment of this histone modification was observed on these genomic regions upon the induction of the TGFβ-dependent EMT and in mesenchymal SW480 cancer cells. More interestingly, METTL3-silencing causes a significant decrease of H3K27me3 enrichment on the same genomic sites (Fig. 7A).

To evaluate whether the observed evidence was due to a different recruitment of Snail on these chromatin sites in dependence on HOTAIR methylation, a ChIP for SNAIL has been performed in control and METTL3-silenced TGFβ-treated hepatocytes as well as in SW480 cancer cells silenced or not for METTL3. Notably, SNAIL interaction with the previously analyzed promoters was not perturbed by METTL3 silencing despite its lack of function (Fig. 7B).

**Fig. 8:**
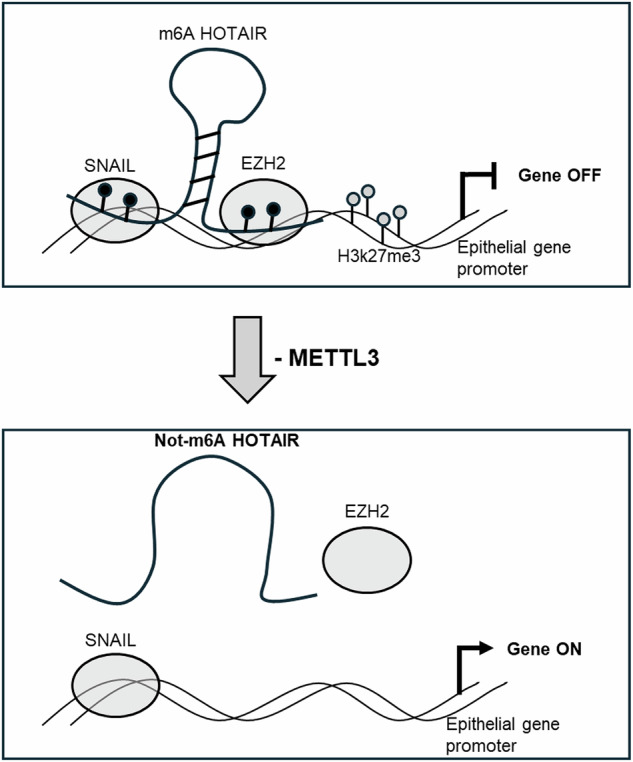
Schematic model illustrating the dominance of m6A modification on HOTAIR function in EMT. Black circles represent m6A and gray circles represent H3K27me3 modifications.

Overall, these data highlight that the m6A modification conditioning specific secondary structure formation is required for the HOTAIR bridging role in the tripartite complex (HOTAIR-SNAIL-EZH2), necessary for the epigenetic repression of epithelial genes triggering EMT (Fig. 8).

## Discussion

This research highlights the epistatic relation of epitranscriptomics on the epigenome. Specifically, with respect to the epigenetic function exerted by the lncRNA HOTAIR in EMT, the m6A epitranscriptomic modification appears to have a dominant role.

m6A modification is catalyzed by the writing complex (composed by METTL3/14) and reversed by the FTO and ALKBH5 demethylases (erasers). Notably, m6A is recognized by different RNA-binding proteins (readers) that exert several molecular functions [15]. Recently, it has also been disclosed the m6A modification effect on miRNAs activity and extracellular vesicle-compartmentalization [14].

Nevertheless, nowadays little is known about the impact of m6A modification on lncRNAs [25]. Despite different lncRNAs have been identified as m6A RNA targets (e.g., Xist [26], HOTAIR [13], MALAT1 [27]) and m6A lncRNAs signatures have been disclosed in different malignancies [28], the impact of this epitranscriptomic modification on their function remains poorly explored yet.

Here, the mechanisms of HOTAIR m6A modification have been disclosed.

Data obtained underlined that: (i) TGFβ-treatment upregulates METTL3 expression and in turn m6A modification, (ii) METTL3 is necessary for EMT induction, cell migration and invasion, (iii) HOTAIR binding domains for SNAIL and EZH2 are m6A-modified, (iv) m6A is propaedeutic for the interaction between HOTAIR and SNAIL/EZH2 and for its bridging role, (v) m6A modification is necessary for the epigenetic role of HOTAIR in the repression of SNAIL-targeted epithelial genes (Fig. 8).

While the regulation of METTL3 expression appears relevant in EMT induction, cell migration/invasion and tumor progression, the complexity of identifying the possible regulators of METTL3 abundance limits the characterization of these molecular dynamics and represents as yet an open field of investigation. Thus, building on a report defining the methylation of HOTAIR on specific sites in breast cancer cells [13], the influence of m6A methylation on the previously described function of HOTAIR in EMT was addressed. Functionally, data indicate that the impairment of m6A modification of the lncHOTAIR results in the reversion of the mesenchymal phenotype and in migration and invasion ability impairment of both non-tumorigenic hepatocytes undergoing TGFβ-induced EMT and mesenchymal carcinoma cells.

Interestingly, the silencing of METTL3 enzyme shows a rescue of the expression levels of SNAIL-HOTAIR-EZH2 targeted epithelial genes without affecting the expression of HOTAIR and SNAIL themselves. Mechanistically, HOTAIR m6A modification impacts its secondary structure and appears pivotal for its capacity to bind to the DNA/RNA binding proteins SNAIL and EZH2 and for the EZH2-dependent H3K27me3 repressive epigenetic modification on E-Cadherin and HNF4α promoters.

These data underline a direct role for SNAIL and EZH2 as direct readers of m6A allowing HOTAIR chromatin association unlike the canonical reader YTHDC1, not involved in these dynamics [13]. Notably, literature data reports additional METTL3-dependent mechanisms contributing to EMT impairment in different tumor cell lines [29, 30] thus the contribution of other players to the complex dynamics occurring in TGFβ-dependent EMT and in HCC cells cannot be ruled out.

With respect to HCC, the impact of m6A has been already reported and, notably, the effectiveness of pharmacological METTL3 inhibition in different animal models of malignancies has been shown to provide an innovative therapeutic approach [31–34]. Our data provide further evidence focusing on the lncRNA HOTAIR and specifically on its activity that highlights the crossroad between epitranscriptomics and epigenetics. In this light HCC warrants further investigation. More in general, it will be essential to consider not only the expression levels of RNA molecules but also their methylation status in the diagnosis of HCC. Conceivably, epitranscriptomic modifications will soon be recognized as playing a mechanistic role in regulating other non-coding RNAs, ultimately shaping specific epigenetic landscapes causal for HCC onset and progression.

## Supplementary information

Supplementary Figure 1

Supplementary Figure 2

Supplementary Figure 3

Supplementary Figure 4

Supplementary Captions

Original Western Blots

## Data Availability

The data generated in this study are available upon request from the corresponding author.

## References

[1] Weng X, Liu H, Ruan J, Du M, Wang L, Mao J, et al. HOTAIR/miR-1277-5p/ZEB1 axis mediates hypoxia-induced oxaliplatin resistance via regulating epithelial-mesenchymal transition in colorectal cancer. Cell Death Discov. 2022;8:310. 35798695 10.1038/s41420-022-01096-0 PMC9263107

[2] Yu F, Chen B, Dong P, Zheng J. HOTAIR epigenetically modulates PTEN expression via MicroRNA-29b: a novel mechanism in regulation of liver fibrosis. Mol Ther. 2017;25:205–17. 28129115 10.1016/j.ymthe.2016.10.015 PMC5363197

[3] Guo B, Cheng Y, Yao L, Zhang J, Lu J, Qi H, et al. LncRNA HOTAIR regulates the lipid accumulation in non-alcoholic fatty liver disease via miR-130b-3p/ROCK1 axis. Cell Signal. 2022;90:110190. 34774989 10.1016/j.cellsig.2021.110190

[4] Hu M, Fu Q, Jing C, Zhang X, Qin T, Pan Y. LncRNA HOTAIR knockdown inhibits glycolysis by regulating miR-130a-3p/HIF1A in hepatocellular carcinoma under hypoxia. Biomed Pharmacother. 2020;125:109703. 32062551 10.1016/j.biopha.2019.109703

[5] Mahpour A, Mullen AC. Our emerging understanding of the roles of long non-coding RNAs in normal liver function, disease, and malignancy. JHEP Rep. 2021;3:100177. 33294829 10.1016/j.jhepr.2020.100177 PMC7689550

[6] Liu Z, Ouyang G, Lu W, Zhang H. Long non-coding RNA HOTAIR promotes hepatocellular carcinoma progression by regulating miR-526b-3p/DHX33 axis. Genes Genom. 2021;43:857–68. 10.1007/s13258-021-01098-9 33843021

[7] Liu Y, Chen X, Chen X, Liu J, Gu H, Fan R, et al. Long non-coding RNA HOTAIR knockdown enhances radiosensitivity through regulating microRNA-93/ATG12 axis in colorectal cancer. Cell Death Dis. 2020;11:175. 32144238 10.1038/s41419-020-2268-8 PMC7060216

[8] Tang B, Bao N, He G, Wang J. Long noncoding RNA HOTAIR regulates autophagy via the miR-20b-5p/ATG7 axis in hepatic ischemia/reperfusion injury. Gene. 2019;686:56–62. 30367982 10.1016/j.gene.2018.10.059

[9] Garbo S, Tripodi M, Battistelli C. lncRNA HOTAIR functions and therapeutic perspectives. Oncoscience. 2022;9:49–51. 36110328 10.18632/oncoscience.563 PMC9469907

[10] Jarroux J, Foretek D, Bertrand C, Gabriel M, Szachnowski U, Saci Z, et al. HOTAIR lncRNA promotes epithelial-mesenchymal transition by redistributing LSD1 at regulatory chromatin regions. EMBO Rep. 2021;22:e50193. 33960111 10.15252/embr.202050193 PMC8366456

[11] Battistelli C, Cicchini C, Santangelo L, Tramontano A, Grassi L, Gonzalez FJ, et al. The Snail repressor recruits EZH2 to specific genomic sites through the enrollment of the lncRNA HOTAIR in epithelial-to-mesenchymal transition. Oncogene. 2017;36:942–55. 27452518 10.1038/onc.2016.260 PMC5318668

[12] Battistelli C, Garbo S, Riccioni V, Montaldo C, Santangelo L, Vandelli A, et al. Design and functional validation of a mutant variant of the LncRNA HOTAIR to counteract snail function in epithelial-to-mesenchymal transition. Cancer Res. 2021;81:103–13. 33158813 10.1158/0008-5472.CAN-20-1764 PMC7611326

[13] Porman AM, Roberts JT, Duncan ED, Chrupcala ML, Levine AA, Kennedy MA, et al. A single N6-methyladenosine site regulates lncRNA HOTAIR function in breast cancer cells. PLoS Biol. 2022;20:e3001885. 36441764 10.1371/journal.pbio.3001885 PMC9731500

[14] Garbo S, D’Andrea D, Colantoni A, Fiorentino F, Mai A, Ramos A, et al. m6A modification inhibits miRNAs’ intracellular function, favoring their extracellular export for intercellular communication. Cell Rep. 2024;43:114369. 38878288 10.1016/j.celrep.2024.114369

[15] Garbo S, Zwergel C, Battistelli C. m6A RNA methylation and beyond—the epigenetic machinery and potential treatment options. Drug Discov Today. 2021;26:2559–74. 34126238 10.1016/j.drudis.2021.06.004

[16] Amicone L, Spagnoli FM, Spath G, Giordano S, Tommasini C, Bernardini S, et al. Transgenic expression in the liver of truncated Met blocks apoptosis and permits immortalization of hepatocytes. EMBO J. 1997;16:495–503. 9034332 10.1093/emboj/16.3.495 PMC1169653

[17] Santangelo L, Giurato G, Cicchini C, Montaldo C, Mancone C, Tarallo R, et al. The RNA-binding protein SYNCRIP is a component of the hepatocyte exosomal machinery controlling MicroRNA sorting. Cell Rep. 2016;17:799–808. 27732855 10.1016/j.celrep.2016.09.031

[18] Bustin SA, Benes V, Garson JA, Hellemans J, Huggett J, Kubista M, et al. The MIQE guidelines: minimum information for publication of quantitative real-time PCR experiments. Clin Chem. 2009;55:611–22. 19246619 10.1373/clinchem.2008.112797

[19] Dominissini D, Moshitch-Moshkovitz S, Salmon-Divon M, Amariglio N, Rechavi G. Transcriptome-wide mapping of N(6)-methyladenosine by m(6)A-seq based on immunocapturing and massively parallel sequencing. Nat Protoc. 2013;8:176–89. 23288318 10.1038/nprot.2012.148

[20] Ponti RD, Armaos A, Vandelli A, Tartaglia GG. CROSSalive: a web server for predicting the in vivo structure of RNA molecules. Bioinformatics. 2020;36:940–1. 31504168 10.1093/bioinformatics/btz666 PMC9883674

[21] Dahm GM, Gubin MM, Magee JD, Techasintana P, Calaluce R, Atasoy U. Method for the isolation and identification of mRNAs, microRNAs and protein components of ribonucleoprotein complexes from cell extracts using RIP-Chip. J Vis Exp. 2012. 10.3791/3851 PMC3490259 23051702

[22] Gupta RA, Shah N, Wang KC, Kim J, Horlings HM, Wong DJ, et al. Long non-coding RNA HOTAIR reprograms chromatin state to promote cancer metastasis. Nature. 2010;464:1071–6. 20393566 10.1038/nature08975 PMC3049919

[23] Li CH, Xiao Z, Tong JH, To KF, Fang X, Cheng AS, et al. EZH2 coupled with HOTAIR to silence MicroRNA-34a by the induction of heterochromatin formation in human pancreatic ductal adenocarcinoma. Int J Cancer. 2017;140:120–9. 27594424 10.1002/ijc.30414

[24] Tsai MC, Manor O, Wan Y, Mosammaparast N, Wang JK, Lan F, et al. Long noncoding RNA as modular scaffold of histone modification complexes. Science. 2010;329:689–93. 20616235 10.1126/science.1192002 PMC2967777

[25] Dinescu S, Ignat S, Lazar AD, Constantin C, Neagu M, Costache M. Epitranscriptomic signatures in lncRNAs and their possible roles in cancer. Genes (Basel). 2019;10. 10.3390/genes10010052 PMC6356509 30654440

[26] Patil DP, Chen CK, Pickering BF, Chow A, Jackson C, Guttman M, et al. m(6)A RNA methylation promotes XIST-mediated transcriptional repression. Nature. 2016;537:369–73. 27602518 10.1038/nature19342 PMC5509218

[27] Liu J, Zhao W, Zhang L, Wang X. The emerging roles of N6-methyladenosine (m6A)-modified long non-coding RNAs in human cancers. Cell Death Discov. 2022;8:255. 35534472 10.1038/s41420-022-01050-0 PMC9085772

[28] Cusenza VY, Tameni A, Neri A, Frazzi R. The lncRNA epigenetics: the significance of m6A and m5C lncRNA modifications in cancer. Front Oncol. 2023;13:1063636. 36969033 10.3389/fonc.2023.1063636 PMC10033960

[29] Yue B, Song C, Yang L, Cui R, Cheng X, Zhang Z, et al. METTL3-mediated N6-methyladenosine modification is critical for epithelial-mesenchymal transition and metastasis of gastric cancer. Mol Cancer. 2019;18:142. 31607270 10.1186/s12943-019-1065-4 PMC6790244

[30] Zhao C, Ling X, Xia Y, Yan B, Guan Q. The m6A methyltransferase METTL3 controls epithelial-mesenchymal transition, migration and invasion of breast cancer through the MALAT1/miR-26b/HMGA2 axis. Cancer Cell Int. 2021;21:441. 34419065 10.1186/s12935-021-02113-5 PMC8380348

[31] Pan Y, Chen H, Zhang X, Liu W, Ding Y, Huang D, et al. METTL3 drives NAFLD-related hepatocellular carcinoma and is a therapeutic target for boosting immunotherapy. Cell Rep Med. 2023;4:101144. 37586322 10.1016/j.xcrm.2023.101144 PMC10439254

[32] Wang L, Yang Q, Zhou Q, Fang F, Lei K, Liu Z, et al. METTL3-m(6)A-EGFR-axis drives lenvatinib resistance in hepatocellular carcinoma. Cancer Lett. 2023;559:216122. 36898427 10.1016/j.canlet.2023.216122

[33] Yankova E, Blackaby W, Albertella M, Rak J, De Braekeleer E, Tsagkogeorga G, et al. Small-molecule inhibition of METTL3 as a strategy against myeloid leukaemia. Nature. 2021;593:597–601. 33902106 10.1038/s41586-021-03536-w PMC7613134

[34] Pomaville M, Chennakesavalu M, Wang P, Jiang Z, Sun HL, Ren P, et al. Small-molecule inhibition of the METTL3/METTL14 complex suppresses neuroblastoma tumor growth and promotes differentiation. Cell Rep. 2024;43:114165. 38691450 10.1016/j.celrep.2024.114165 PMC11181463

