# Supplementary figures and images for: HOTAIR requires epitranscriptomic modification to exert its pivotal epigenetic role in Epithelial to Mesenchymal Transition
Source: Cell Death Dis. 2025 Oct 24;16(1):753. doi: 10.1038/s41419-025-08099-6 (PMC12552435; doi:10.1038/s41419-025-08099-6)

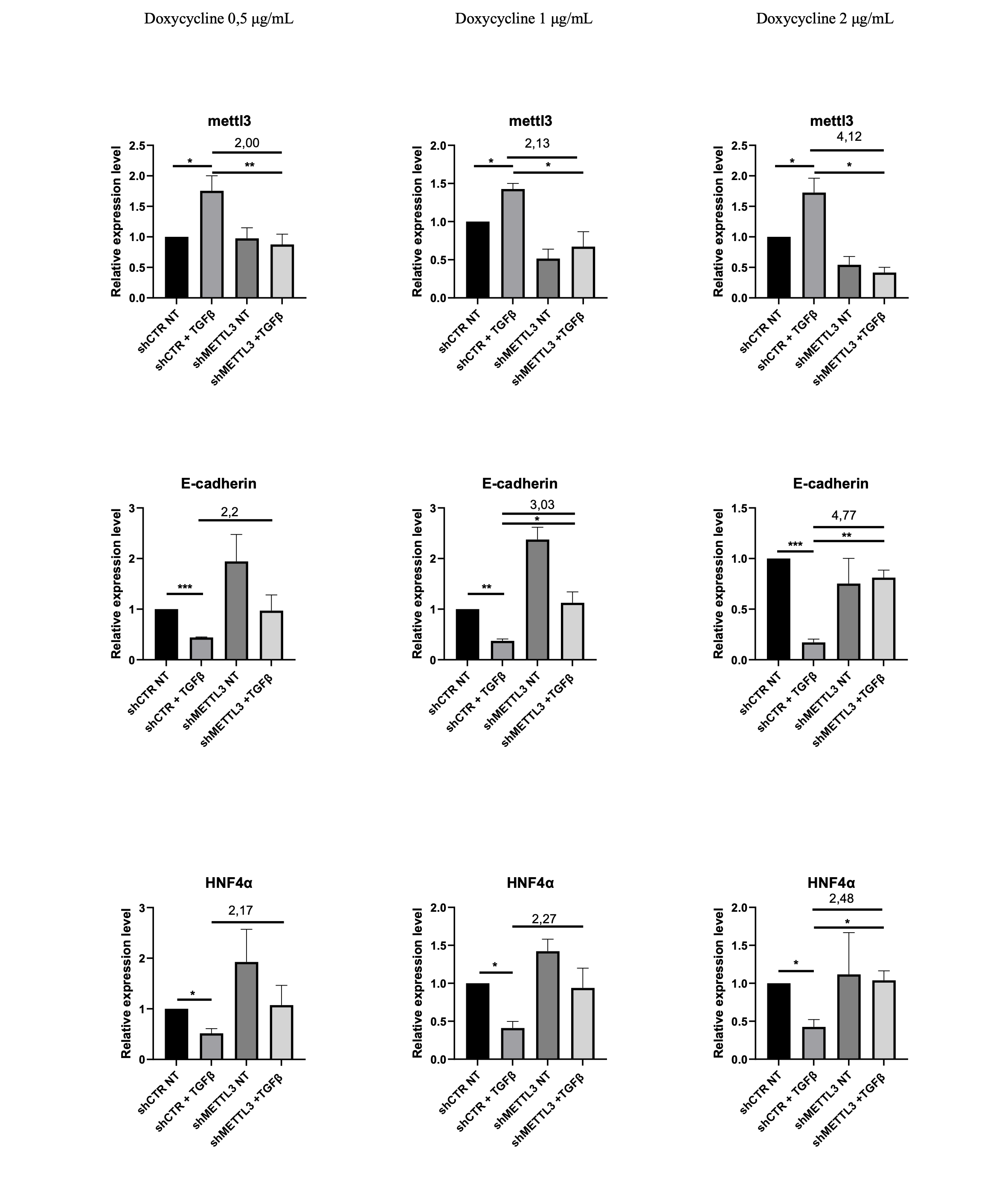

Supplement: Supplementary file 1 — Supplementary Figure 1 [file 41419_2025_8099_MOESM1_ESM.png]

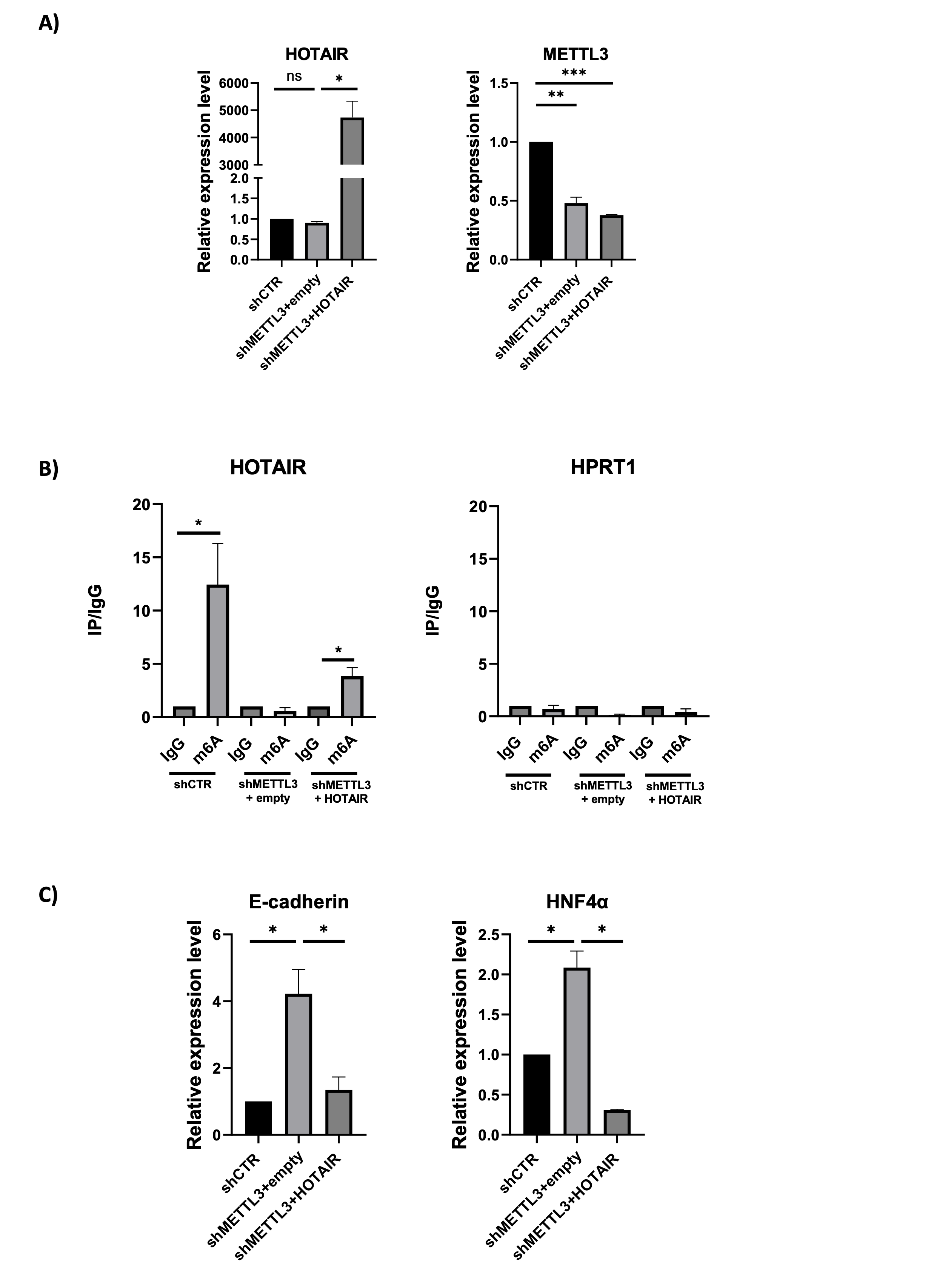

Supplement: Supplementary file 2 — Supplementary Figure 2 [file 41419_2025_8099_MOESM2_ESM.png]

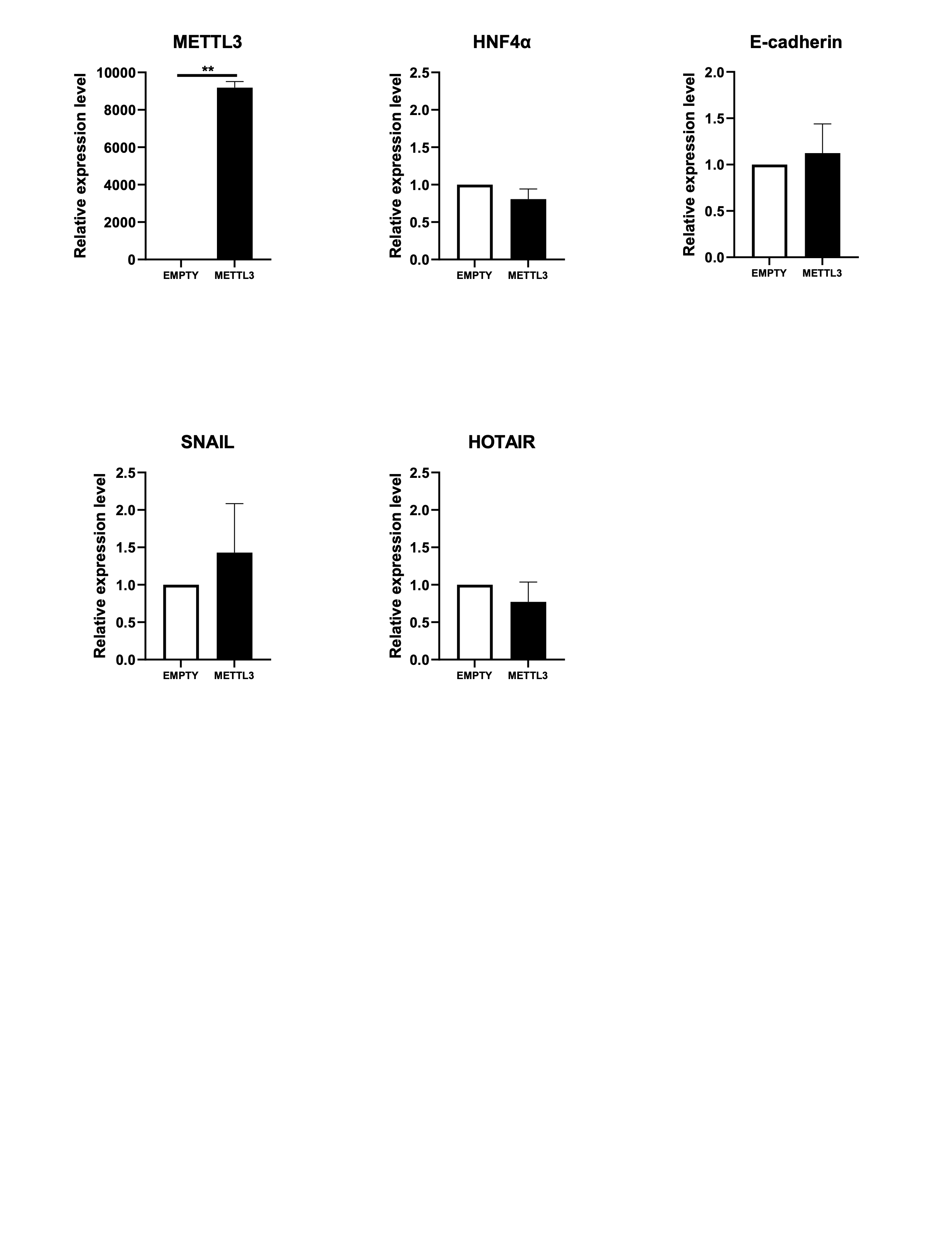

Supplement: Supplementary file 3 — Supplementary Figure 3 [file 41419_2025_8099_MOESM3_ESM.png]

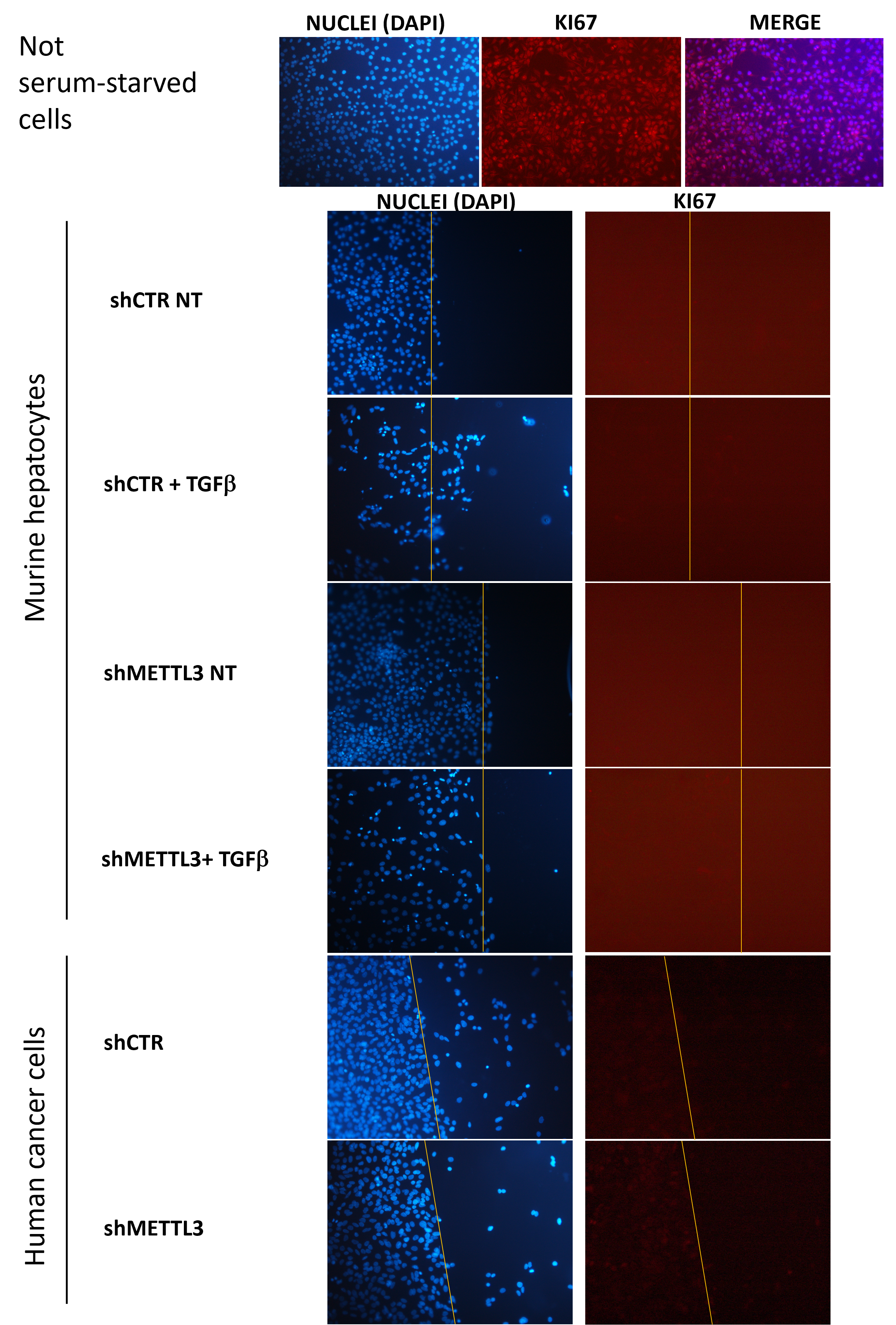

Supplement: Supplementary file 4 — Supplementary Figure 4 [file 41419_2025_8099_MOESM4_ESM.png]
