# Supplementary material for: HOTAIR requires epitranscriptomic modification to exert its pivotal epigenetic role in Epithelial to Mesenchymal Transition
Source: Cell Death Dis. 2025 Oct 24;16(1):753. doi: 10.1038/s41419-025-08099-6 (PMC12552435; doi:10.1038/s41419-025-08099-6)
Supplement: Supplementary file 5 — Supplementary Captions [file 41419_2025_8099_MOESM5_ESM.pptx]

## Slide 1
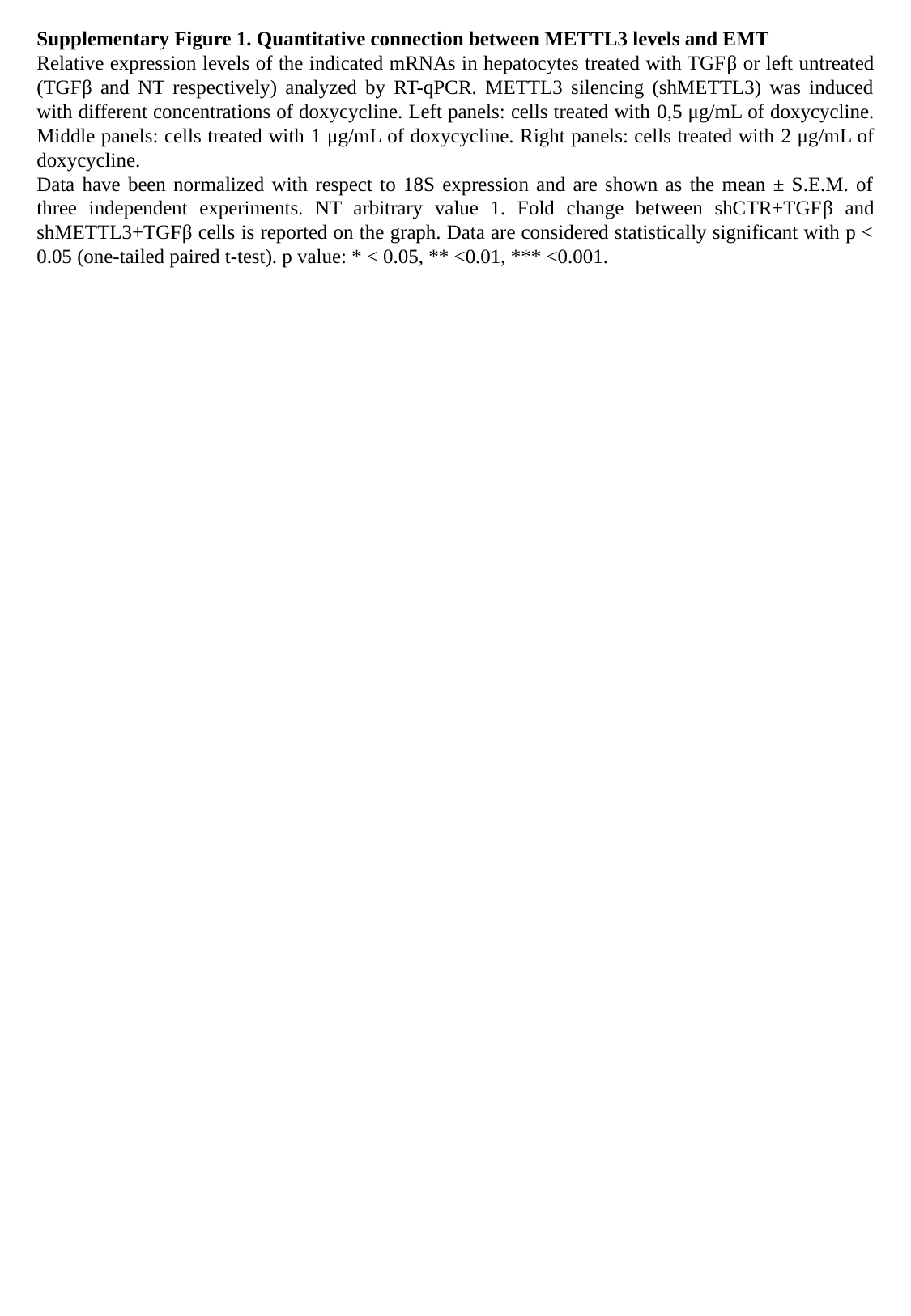

Supplementary Figure 1. Quantitative connection between METTL3 levels and EMT
Relative expression levels of the indicated mRNAs in hepatocytes treated with TGFβ or left untreated (TGFβ and NT respectively) analyzed by RT-qPCR. METTL3 silencing (shMETTL3) was induced with different concentrations of doxycycline. Left panels: cells treated with 0,5 μg/mL of doxycycline. Middle panels: cells treated with 1 μg/mL of doxycycline. Right panels: cells treated with 2 μg/mL of doxycycline.
Data have been normalized with respect to 18S expression and are shown as the mean ± S.E.M. of three independent experiments. NT arbitrary value 1. Fold change between shCTR+TGFβ and shMETTL3+TGFβ cells is reported on the graph. Data are considered statistically significant with p < 0.05 (one-tailed paired t-test). p value: * < 0.05, ** <0.01, *** <0.001.

## Slide 2
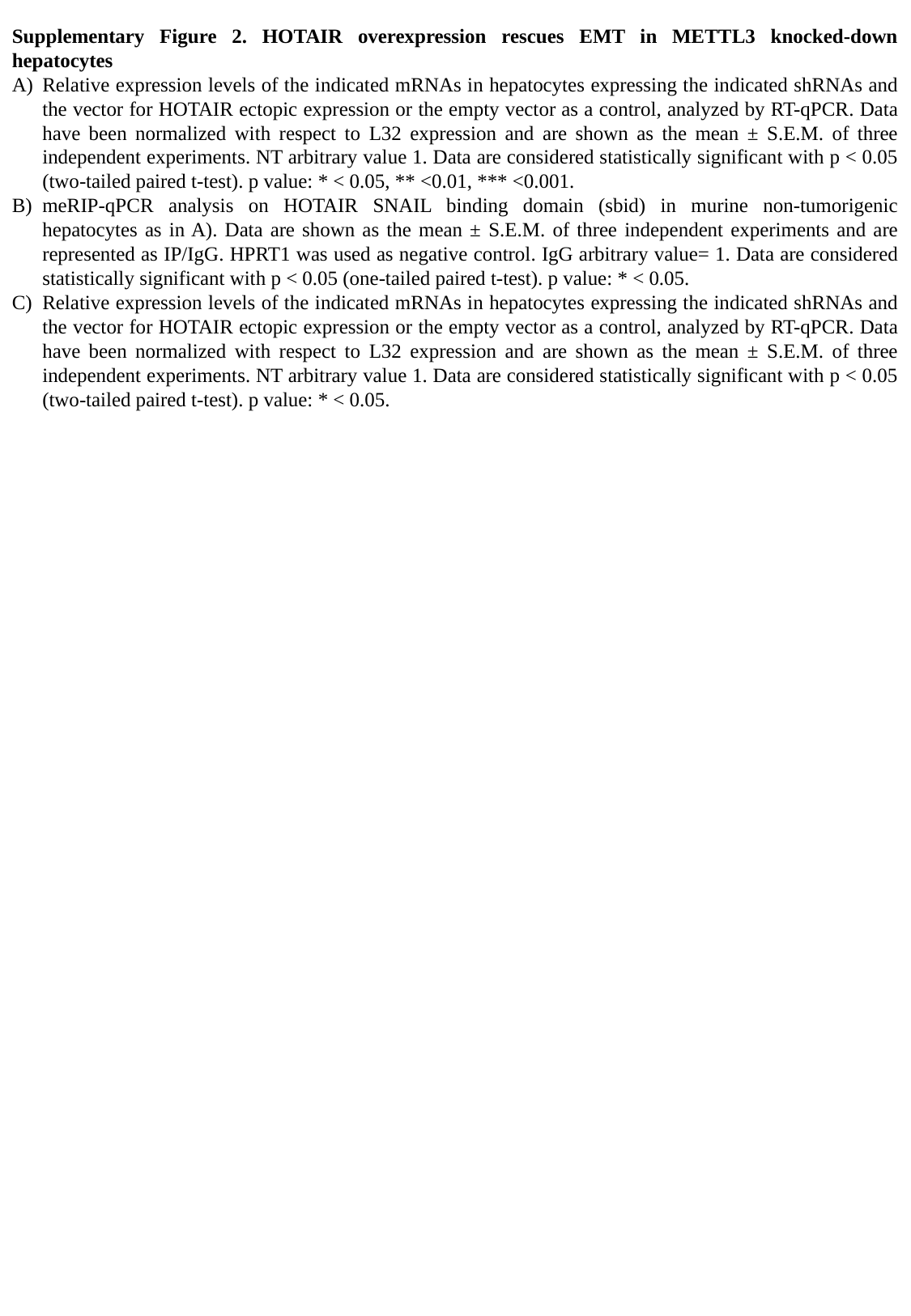

Supplementary Figure 2. HOTAIR overexpression rescues EMT in METTL3 knocked-down hepatocytes
Relative expression levels of the indicated mRNAs in hepatocytes expressing the indicated shRNAs and the vector for HOTAIR ectopic expression or the empty vector as a control, analyzed by RT-qPCR. Data have been normalized with respect to L32 expression and are shown as the mean ± S.E.M. of three independent experiments. NT arbitrary value 1. Data are considered statistically significant with p < 0.05 (two-tailed paired t-test). p value: * < 0.05, ** <0.01, *** <0.001.
meRIP-qPCR analysis on HOTAIR SNAIL binding domain (sbid) in murine non-tumorigenic hepatocytes as in A). Data are shown as the mean ± S.E.M. of three independent experiments and are represented as IP/IgG. HPRT1 was used as negative control. IgG arbitrary value= 1. Data are considered statistically significant with p < 0.05 (one-tailed paired t-test). p value: * < 0.05.
Relative expression levels of the indicated mRNAs in hepatocytes expressing the indicated shRNAs and the vector for HOTAIR ectopic expression or the empty vector as a control, analyzed by RT-qPCR. Data have been normalized with respect to L32 expression and are shown as the mean ± S.E.M. of three independent experiments. NT arbitrary value 1. Data are considered statistically significant with p < 0.05 (two-tailed paired t-test). p value: * < 0.05.

## Slide 3
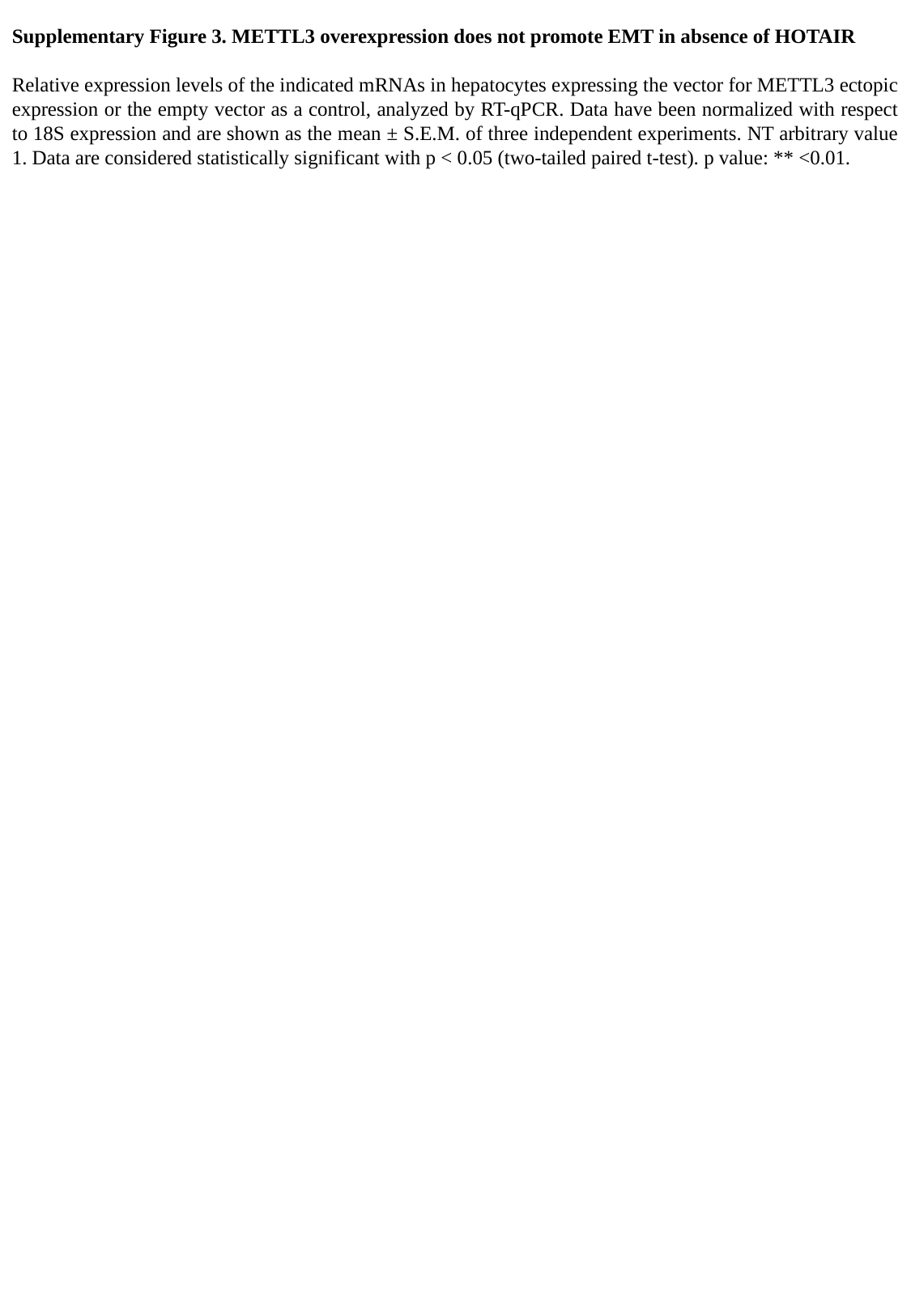

Supplementary Figure 3. METTL3 overexpression does not promote EMT in absence of HOTAIR
Relative expression levels of the indicated mRNAs in hepatocytes expressing the vector for METTL3 ectopic expression or the empty vector as a control, analyzed by RT-qPCR. Data have been normalized with respect to 18S expression and are shown as the mean ± S.E.M. of three independent experiments. NT arbitrary value 1. Data are considered statistically significant with p < 0.05 (two-tailed paired t-test). p value: ** <0.01.

## Slide 4
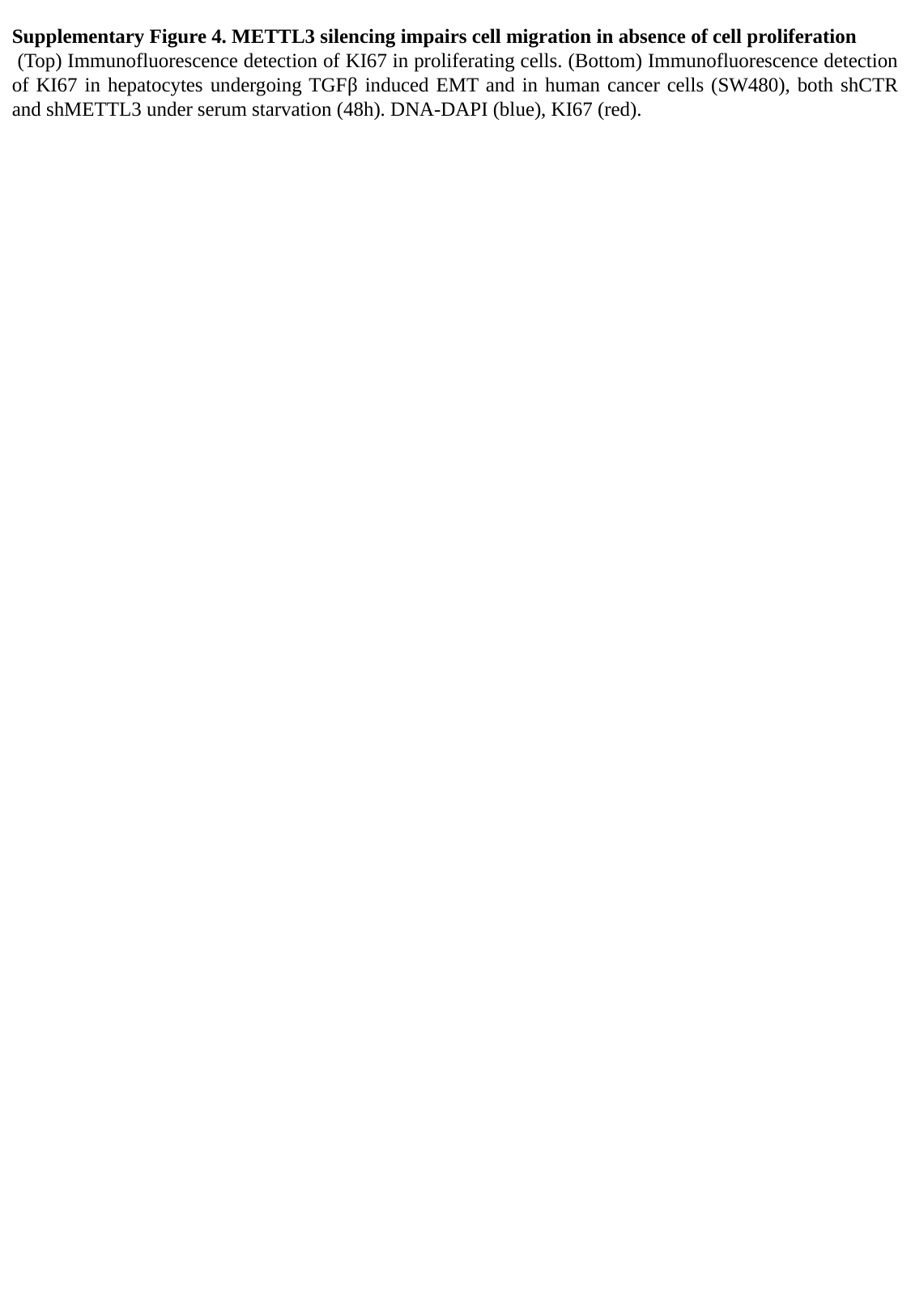

Supplementary Figure 4. METTL3 silencing impairs cell migration in absence of cell proliferation
 (Top) Immunofluorescence detection of KI67 in proliferating cells. (Bottom) Immunofluorescence detection of KI67 in hepatocytes undergoing TGFβ induced EMT and in human cancer cells (SW480), both shCTR and shMETTL3 under serum starvation (48h). DNA-DAPI (blue), KI67 (red).
