# Supplementary material for: HOTAIR requires epitranscriptomic modification to exert its pivotal epigenetic role in Epithelial to Mesenchymal Transition
Source: Cell Death Dis. 2025 Oct 24;16(1):753. doi: 10.1038/s41419-025-08099-6 (PMC12552435; doi:10.1038/s41419-025-08099-6)
Supplement: Supplementary file 6 — Original Western Blots [file 41419_2025_8099_MOESM6_ESM.pptx]

## Slide 1
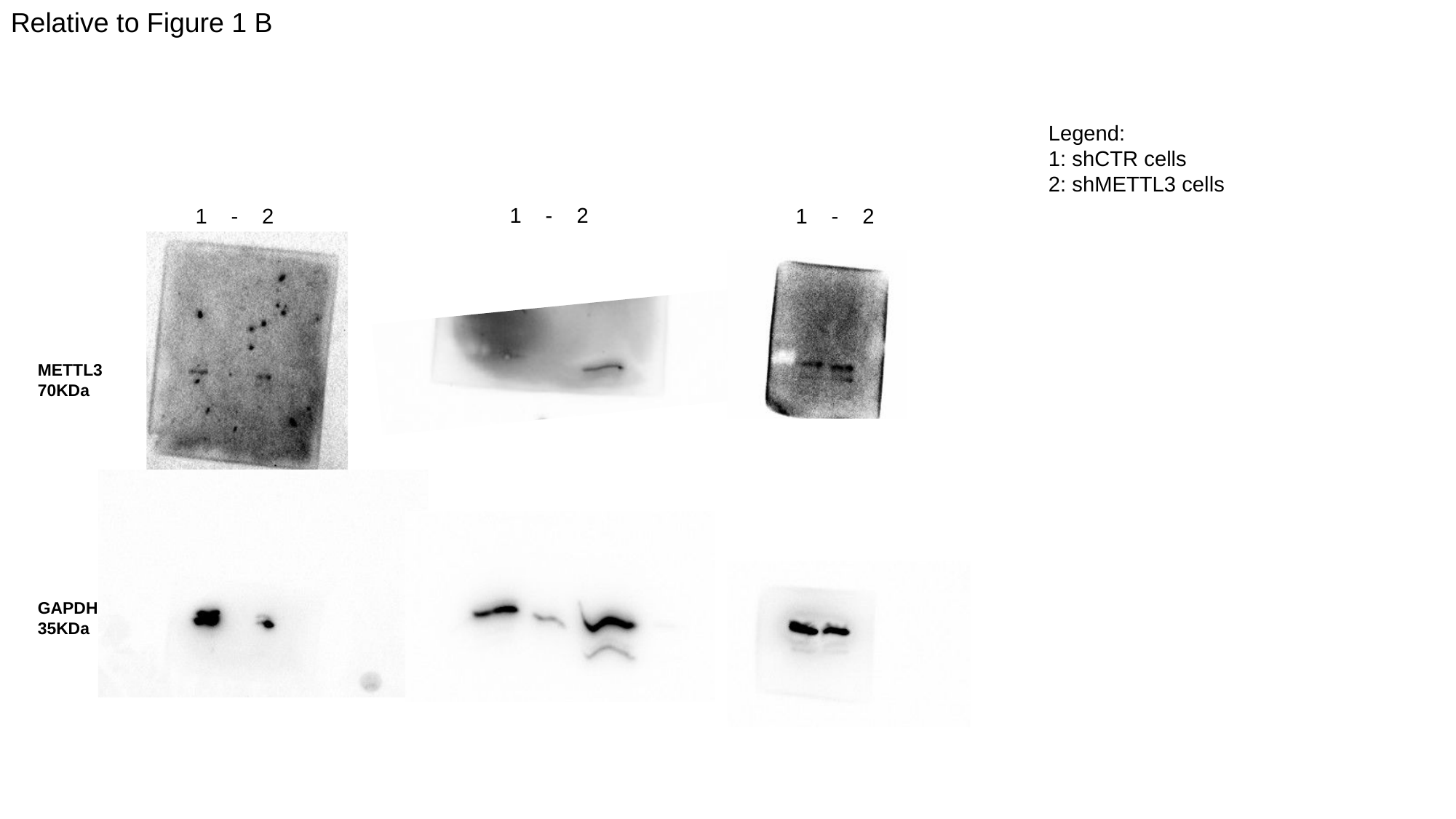

Relative to Figure 1 B
Legend:
1: shCTR cells
2: shMETTL3 cells
1 - 2
1 - 2
1 - 2
METTL3
70KDa
GAPDH
35KDa

## Slide 2
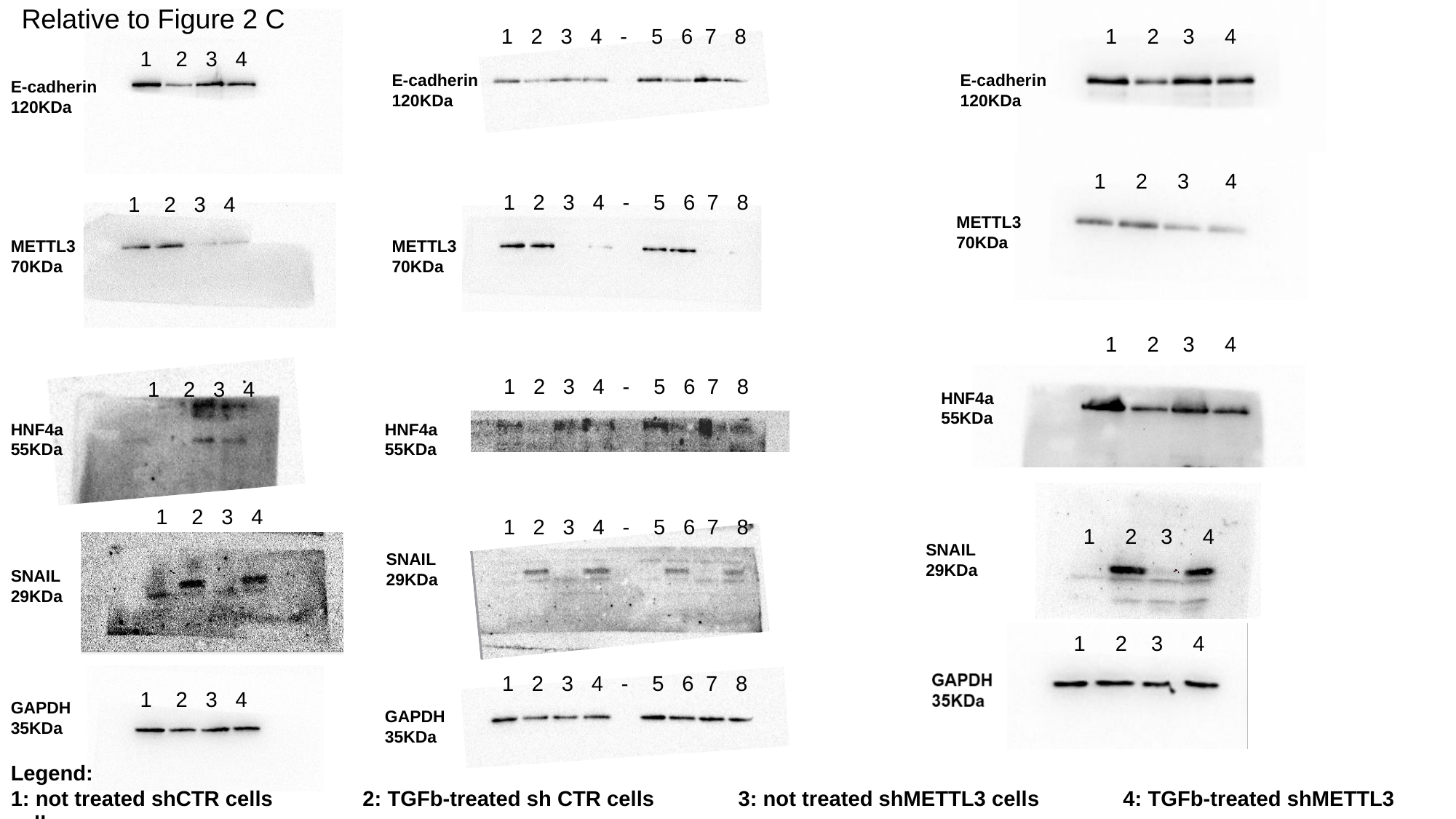

Relative to Figure 2 C
1 2 3 4
1 2 3 4 - 5 6 7 8
1 2 3 4
E-cadherin
120KDa
E-cadherin
120KDa
E-cadherin
120KDa
1 2 3 4
1 2 3 4 - 5 6 7 8
1 2 3 4
METTL3
70KDa
METTL3
70KDa
METTL3
70KDa
1 2 3 4
1 2 3 4 - 5 6 7 8
1 2 3 4
HNF4a
55KDa
HNF4a
55KDa
HNF4a
55KDa
1 2 3 4
1 2 3 4 - 5 6 7 8
1 2 3 4
SNAIL
29KDa
SNAIL
29KDa
SNAIL
29KDa
1 2 3 4
1 2 3 4 - 5 6 7 8
1 2 3 4
GAPDH
35KDa
GAPDH
35KDa
Legend:
1: not treated shCTR cells 2: TGFb-treated sh CTR cells 3: not treated shMETTL3 cells 4: TGFb-treated shMETTL3 cells

## Slide 3
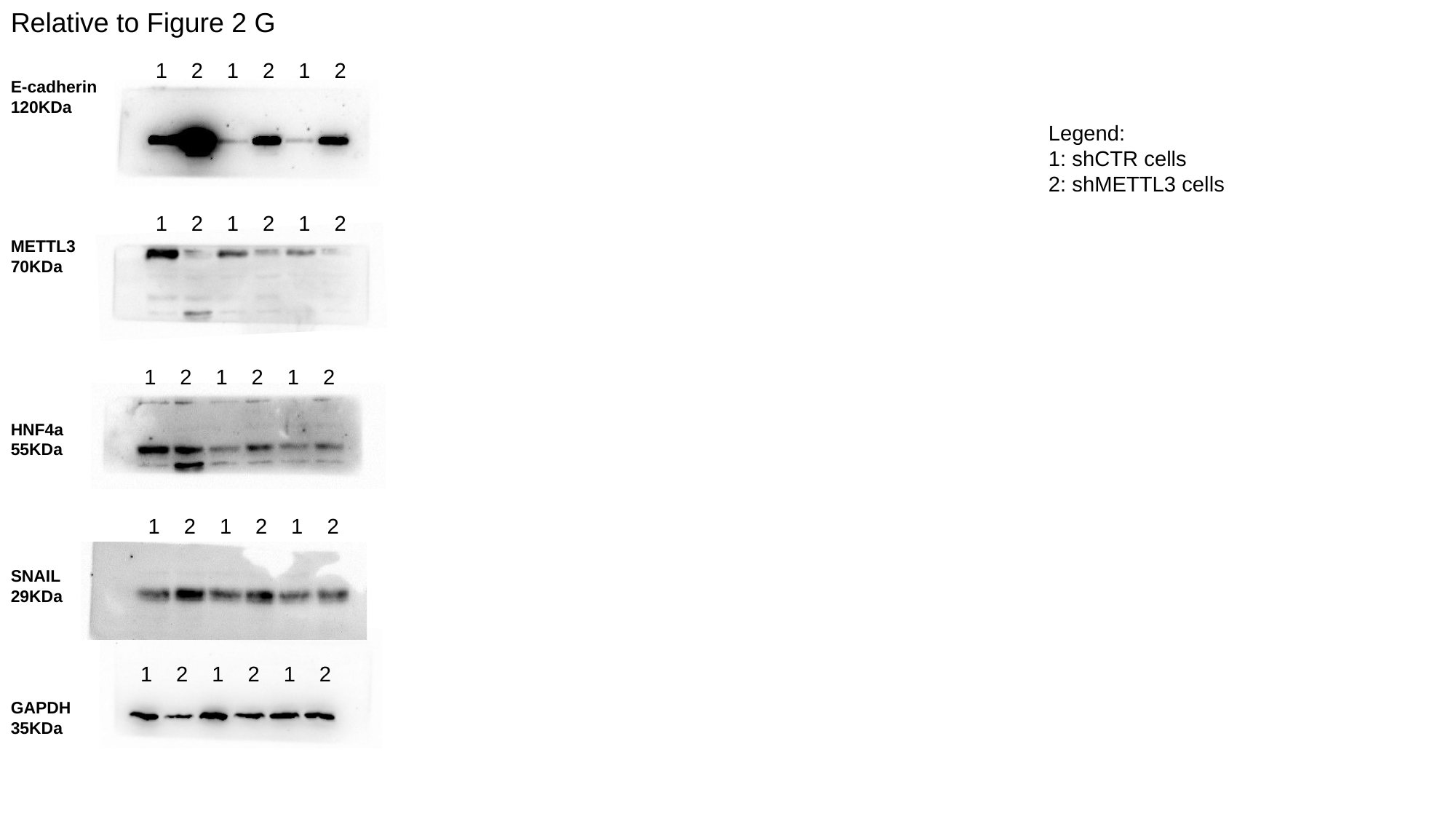

Relative to Figure 2 G
1 2 1 2 1 2
E-cadherin
120KDa
Legend:
1: shCTR cells
2: shMETTL3 cells
1 2 1 2 1 2
METTL3
70KDa
1 2 1 2 1 2
HNF4a
55KDa
1 2 1 2 1 2
SNAIL
29KDa
1 2 1 2 1 2
GAPDH
35KDa

## Slide 4
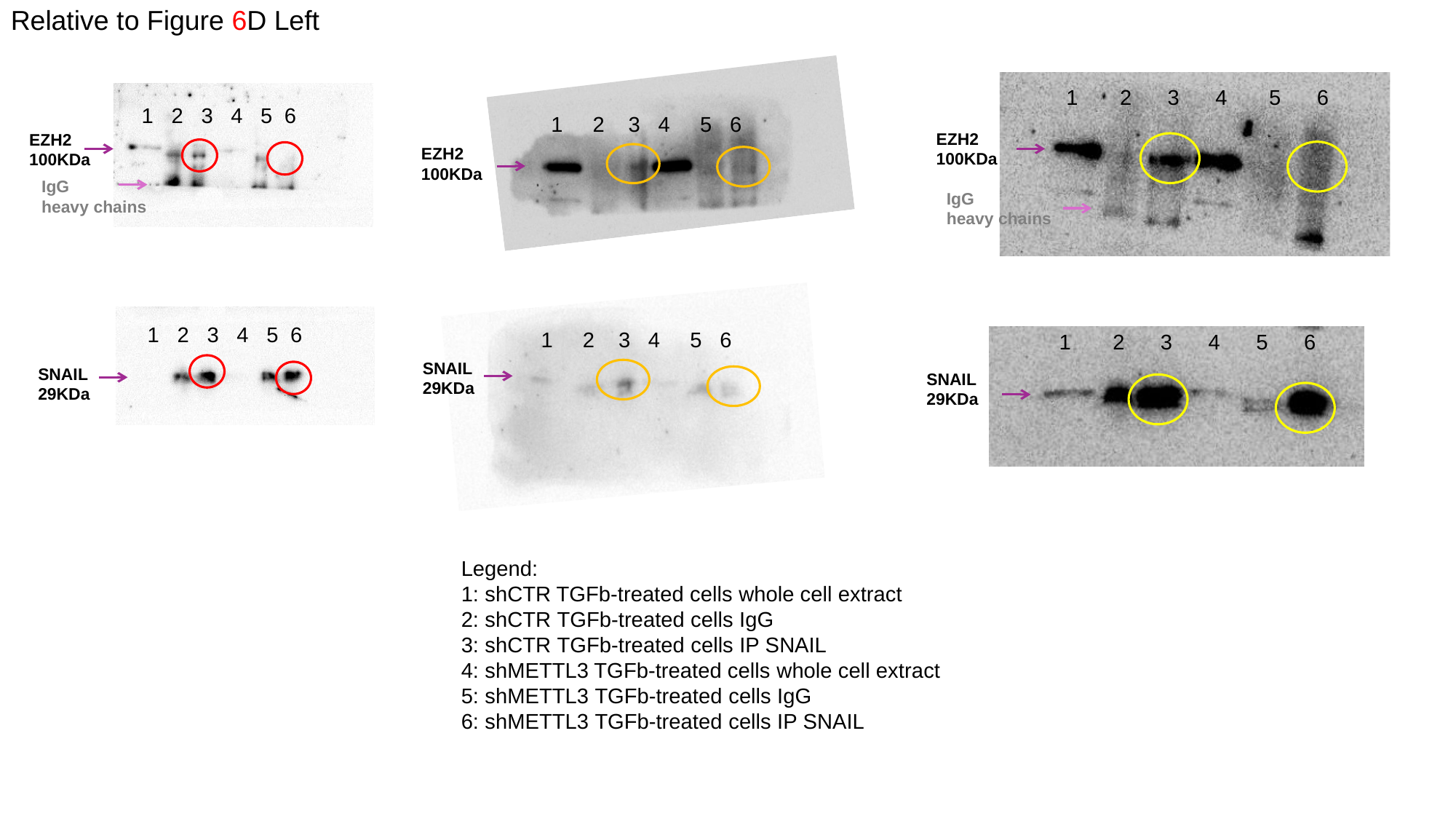

Relative to Figure 6D Left
1 2 3 4 5 6
1 2 3 4 5 6
1 2 3 4 5 6
EZH2
100KDa
EZH2
100KDa
EZH2
100KDa
IgG
heavy chains
IgG
heavy chains
1 2 3 4 5 6
1 2 3 4 5 6
1 2 3 4 5 6
SNAIL
29KDa
SNAIL
29KDa
SNAIL
29KDa
Legend:
1: shCTR TGFb-treated cells whole cell extract
2: shCTR TGFb-treated cells IgG
3: shCTR TGFb-treated cells IP SNAIL
4: shMETTL3 TGFb-treated cells whole cell extract
5: shMETTL3 TGFb-treated cells IgG
6: shMETTL3 TGFb-treated cells IP SNAIL

## Slide 5
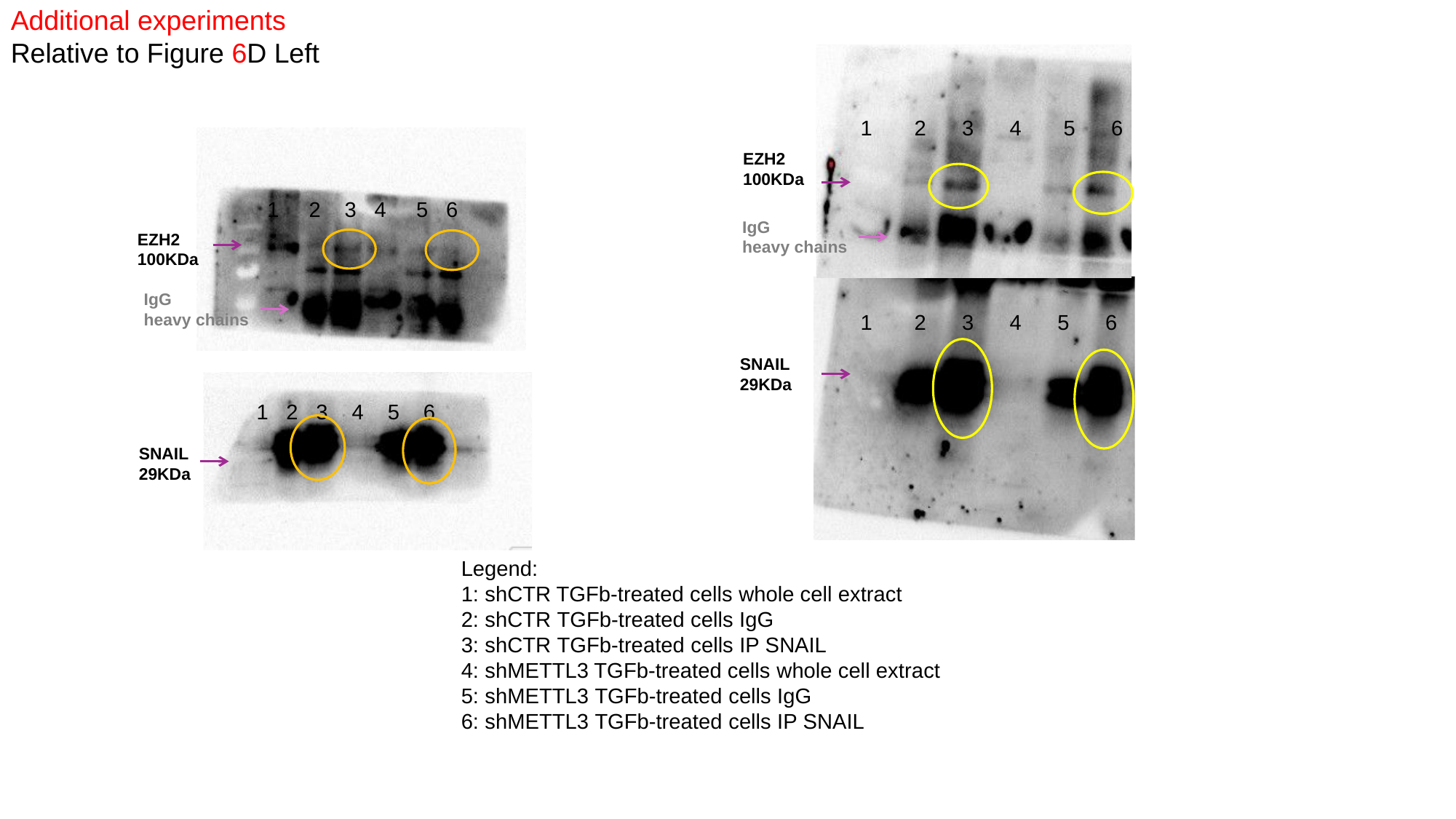

Additional experiments Relative to Figure 6D Left
1 2 3 4 5 6
EZH2
100KDa
1 2 3 4 5 6
IgG
heavy chains
EZH2
100KDa
IgG
heavy chains
1 2 3 4 5 6
SNAIL
29KDa
1 2 3 4 5 6
SNAIL
29KDa
Legend:
1: shCTR TGFb-treated cells whole cell extract
2: shCTR TGFb-treated cells IgG
3: shCTR TGFb-treated cells IP SNAIL
4: shMETTL3 TGFb-treated cells whole cell extract
5: shMETTL3 TGFb-treated cells IgG
6: shMETTL3 TGFb-treated cells IP SNAIL

## Slide 6
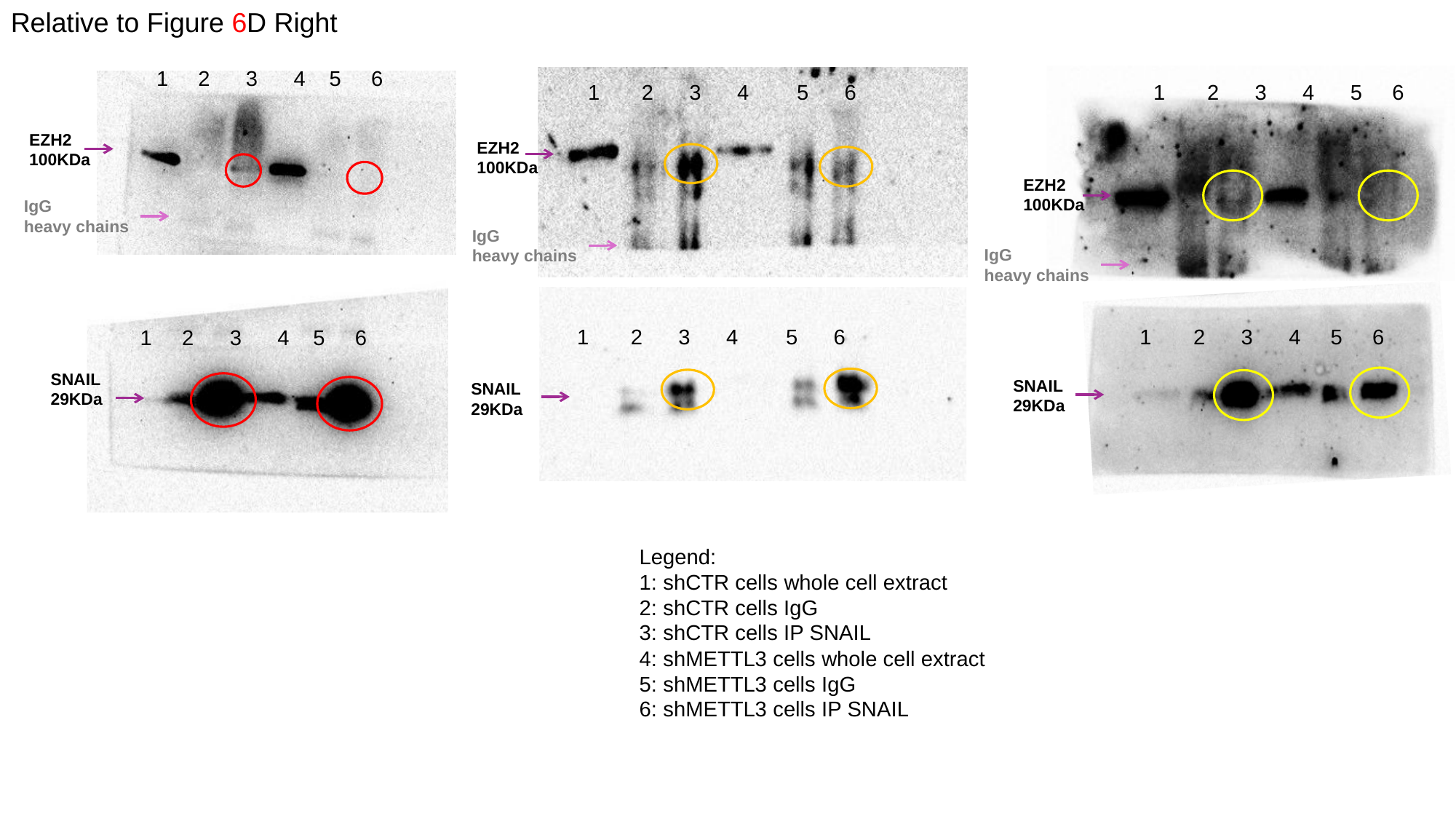

Relative to Figure 6D Right
1 2 3 4 5 6
1 2 3 4 5 6
1 2 3 4 5 6
EZH2
100KDa
EZH2
100KDa
EZH2
100KDa
IgG
heavy chains
IgG
heavy chains
IgG
heavy chains
1 2 3 4 5 6
1 2 3 4 5 6
1 2 3 4 5 6
SNAIL
29KDa
SNAIL
29KDa
SNAIL
29KDa
Legend:
1: shCTR cells whole cell extract
2: shCTR cells IgG
3: shCTR cells IP SNAIL
4: shMETTL3 cells whole cell extract
5: shMETTL3 cells IgG
6: shMETTL3 cells IP SNAIL

## Slide 7
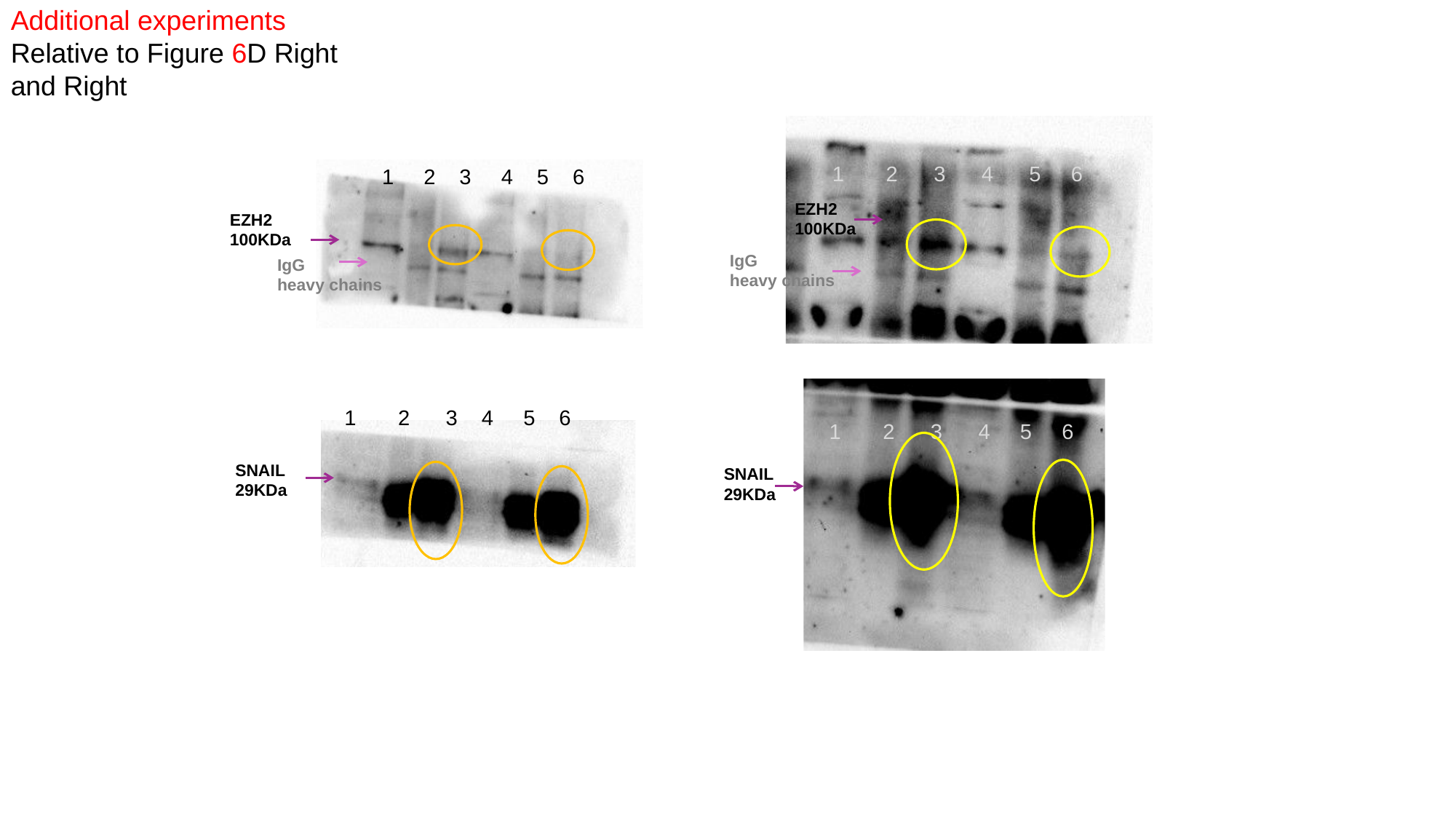

Additional experiments Relative to Figure 6D Right and Right
1 2 3 4 5 6
1 2 3 4 5 6
EZH2
100KDa
EZH2
100KDa
IgG
heavy chains
IgG
heavy chains
1 2 3 4 5 6
1 2 3 4 5 6
SNAIL
29KDa
SNAIL
29KDa
